# Optimization of a bionic end-effector for automated cherry tomato harvesting using Response Surface Methodology and Box-Behnken design

**DOI:** 10.1371/journal.pone.0352701

**Published:** 2026-08-14

**Authors:** Huaibei Xie, Yuting Miao, Qingliang Zi, Deyi Kong

**Affiliations:** 1 School of Mechatronics Engineering, Anhui University of Science and Technology, Huainan, Anhui, China; 2 Anhui Key Laboratory of Mine Intelligent Equipment and Technology, Anhui University of Science and Technology, Huainan, China; 3 Institute of Intelligent Machines, Hefei Institutes of Physical Science, Chinese Academy of Sciences, Hefei, Anhui, China; China University of Mining and Technology, CHINA

## Abstract

Harvesting cherry tomatoes is a labor-intensive and time-consuming endeavor. The implementation of robots for this task represents an effective solution. The end-effector is a vital component of the harvesting robot, essential for automated picking of cherry tomatoes. This study introduces an end-effector with three grippers, modeled on the structure and mechanics of the phalangeal chain found in vultures’ legs, to enhance the efficiency of harvesting robots. To optimize the bionic end-effector’s structural parameters for cherry tomato picking, Response Surface Methodology (RSM) with a Box Behnken Design (BBD) and a desirability-based multi-response optimization strategy was employed. Experiments varied four process parameters at three levels: Width of the Distal Phalanges and Middle Phalanges (WDMP), Thickness of the Distal Phalanges and Middle Phalanges (TDMP), Angle at the End of the Distal Phalanx (AEDP), and Distance between the Heel Phalanges and the Central Axis of the Fixed Seat (DPAS). The maximum inscribed circle diameters of the middle phalanges (DCMP) and distal phalanges (DCDP) were selected as surrogate geometric responses because they represent, respectively, the compressive clearance related to fruit damage risk and the distal enclosure space related to fruit retention stability. The optimal configuration was identified as WDMP 16 mm, TDMP 5 mm, AEDP 90°, and DPAS 7.5 mm. Furthermore, experimental validation of the optimized bionic end-effector demonstrated a harvesting success rate of at least 88% within the tested equatorial-diameter range. This research provides valuable insights and a theoretical basis for future designs of end-effectors for cherry tomato harvesting.

## Introduction

Cherry tomatoes are widely consumed worldwide, with substantial annual demand and extensive cultivation leading to significant global output [1]. However, cherry tomato harvesting is still largely performed manually, leading to high labor costs and intensive effort. Like other agricultural industries, it faces growing sustainability challenges due to labor shortages and rising expenses [2]. The implementation of harvesting robots is essential for addressing these contemporary challenges [3]. While fruit recognition, positioning, and arm motion planning strongly influence harvesting robot performance, the end-effector remains the most critical component [4]. While stem characteristics influence success, the irregular shapes and orientations of fruit in complex environments present significant challenges [5]. Developing a high-performance end-effector is crucial for enhancing the efficiency of cherry tomato harvesting robots.

Extensive research on end-effector designs has led to successful applications in harvesting various fruits, including apples [6], cherries [7], strawberries [8], citrus [9], grapes [10], tomatoes [11,12], cucumbers [13], and peppers [14,15]. For instance, Fujinaga et al. [16] developed a suction-cutting device for large tomato harvesting, achieving a 52.4% success rate, though obstacles around the fruit posed challenges. Xiong et al. [17] created a strawberry-harvesting robot with a cable-driven gripper and internal container, achieving a 53.6% success rate with an average cycle time of 7.5 seconds per fruit. Williams et al. [18] introduced a multi-arm kiwifruit harvesting robot with clamping grippers, achieving an 84% success rate and a cycle time of 5.5 seconds per fruit. Advances in bionic technology have inspired high-performance end-effectors based on animal mechanics. Wang et al. [19] designed a citrus-harvesting end-effector mimicking a snake’s bite, achieving a 74% success rate in natural environments. Roshanianfard et al. [20] developed a five-fingered anthropomorphic end-effector for pumpkin harvesting, with a 79% success rate and the ability to handle crops within a 76.2 mm –265 mm radius.

Our research group designed a cherry tomato-harvesting bionic end-effector inspired by the phalangeal chain mechanics of Serica orientalis Motschulsky, achieving a 76% success rate, though with high fruit damage [21]. This earlier result indicated that stable enclosure and damage control still needed improvement during fruit detachment. Li et al. [22] designed four biomimetic flexible end-effectors, with the Vicia faba L. flexible model showing the lowest damage rate of 1.7%. Federica et al. [23] developed avian-inspired torsion- and bending-resistant structures to enhance mechanical robustness, but the added stiffness and structural complexity may hinder their adaptability in fruit harvesting scenarios. Zang et al. [24] developed a self-adaptive envelope end-effector capable of engulfing and releasing objects through adjustments in torus skin. Liu et al. [25] designed a self-lockable constant-force compliant gripper that ensured stable grasping without active force control, thereby improving reliability and reducing control complexity. While bionic end-effectors demonstrate strong harvesting capabilities, challenges remain. Simple mechanical designs lack adaptability for diverse fruit shapes and risk causing damage. Flexible mechanisms often create oversized clamping areas, complicating precise positioning. These issues are exacerbated when obstacles such as branches exacerbate these issues, further increasing the likelihood of harvesting failure.

To prevent fruit from slipping out of the bionic end-effector and enhance the picking success rate, a bio-inspired phalangeal chain gripper, modeled after vulture leg mechanics, was designed and optimized. Compared with our earlier cherry tomato gripper, the present vulture-inspired design was intended to improve mechanical performance through coordinated underactuated motion of the proximal, middle, and distal phalanges, enhance adaptability by allowing progressive self-wrapping under fruit-position deviation or partial enclosure, and reduce damage by removing talon-like features and optimizing the contact geometry of the grasping area. The optimization employed Response Surface Methodology (RSM) with a Box Behnken Design (BBD) approach to refine key structural parameters for effective robotic cherry tomato harvesting. Four parameters—the width of the distal phalanges and middle phalanges, the thickness of the distal phalanges and middle phalanges, the angle at the end of the distal phalanx, and the distance between the heel phalanges and the central axis of the fixed seat—were tested at three levels to analyze their effects on fruit retention. Among the two response variables, DCMP was used to characterize the compressive clearance between the gripper and fruit and thus the potential squeezing damage, whereas DCDP was used to characterize the distal enclosure space and thus the anti-slip anti-interference capability during detachment. Therefore, the optimization objective was defined as a multi-criteria compromise between damage reduction and stable retention, rather than the maximization of a single response. A quadratic model and expectation function were established using RSM-BBD, and their reliability was confirmed through experiments. The optimal parameter combination was identified, and experiments validated the harvesting performance of the optimized end-effector. Additionally, by adjusting these parameters, the model can be adapted to ensure high success rates for various fruit types.

This study investigates an end-effector inspired by the legs of vultures, utilizing a bio-inspired phalangeal chain gripper. Experimental design and parameter optimization for the bionic end-effector are detailed, starting with a description of the cherry tomato samples. Accordingly, the key contribution of this study is not only parameter optimization, but also clarification of how the redesigned phalangeal-chain structure stabilizes grasping, improves tolerance to partial enclosure, and lowers the risks of fruit slippage and bruising during harvesting.

This study focuses on a vulture-inspired end-effector featuring a bio-inspired phalangeal chain gripper. It presents experimental design and parameter optimization, starting with a description of the cherry tomato samples. Section 2 covers the gripper design, the overall end-effector structure, and the main equipment and methods used for cherry tomato harvesting. Section 3 provides a comprehensive account of our experimental methodologies and results. Subsequently, in Section 4, we discuss how different parameters associated with the bionic end-effector influence evaluation factors pertinent to fruit harvesting outcomes based on our experimental findings. Finally, conclusions drawn from this research are presented in Section 5.

## Materials and methods

### Cherry tomato samples

Fresh “HONGYAN” cherry tomatoes were used for picking experiments. All tests to optimize the bionic end-effector’s performance were conducted during the maturity stage of the tomatoes. The cherry tomatoes used for performance testing had an equatorial diameter of 25.88 mm ± 1.75 mm and a mass of 9.89 g ± 1.92 g. Previous studies on fruit detachment mechanics indicate that developmental stage and cultivar can influence detachment-related behavior [26]. Accordingly, in the present study fruits were selected at a similar maturity stage and within a relatively narrow size/mass distribution to reduce biological variability during structural optimization. Therefore, these sample descriptors are reported to define the tested fruit range rather than claim that fruit physical characteristics have negligible effects on picking success or separation force.

### Physiological structure characteristics and biogenic source analysis of the bio-inspired phalangeal chain gripper

**Fig 1:**
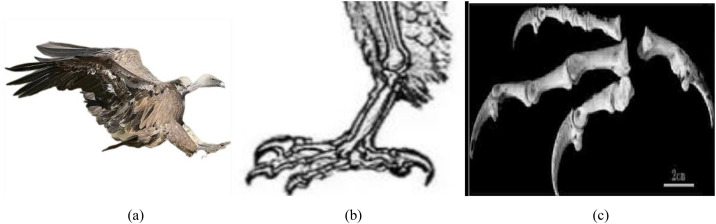
The structure of a vulture’s leg and its prey capture process. (a) Catching prey; (b) The physiological anatomy of legs, (c) The Structure of phalangeal chain of toes.

As shown in Fig 1(a), the process by which a vulture uses its legs to capture prey can be divided into three stages: the first stage involves the toes spreading and grasping the prey, the second stage involves holding the prey tightly, and the third stage involves releasing the prey. In the first stage, the phalanges of the vulture’s toes open around the metacarpal bone and align with the prey. The second stage mainly involves the prey being grasped tightly by the phalanges of the vulture’s toes. The third stage is the process of releasing the prey by the phalanges of the vulture’s toes. Based on the above process, the process of vulture hunting prey can be roughly divided into five steps: extending, aligning, opening, grasping, and releasing, which are very similar to the process of picking cherry tomatoes by the harvesting manipulator. The extension and alignment actions can be completed by the harvesting manipulator of the picking robot, while the opening, grasping, and releasing actions are performed by the picking end-effector.

In accordance with the anatomical structure of the vulture’s legs as described in literature [27], the vulture’s legs are mainly composed of epidermis, metacarpal bone, toes, ligament, flexor tendon, and muscle, wherein the toe is composed of multiple phalanges (Fig 1b). According to the distribution of the phalanges in the vulture’s legs (Fig 1c), the vulture’s leg has four toes: the inner toe, middle toe, outer toe, and hind toe. The number of phalanges in each toe is unequal, and their lengths are different. The end of each toe has a curved talon.

By observing the leg structure of vultures, it can be ascertained that the legs of vultures are naturally evolved hand-foot reusable mechanisms. This distinctive physiological structural feature enables vultures to select appropriate grasping patterns based on the size of the target object, thereby enhancing the success rate of capturing the target, which is significantly different from the functional attributes of the phalangeal chain structure characteristics of animals such as Serica orientalis Motschulsky [28]. Through observing the process of vultures’ legs preying on targets, it was discovered that for larger objects like hares, the legs of vultures adjust the positions of the middle phalanges of each toe to envelop the target object. For smaller objects like branches, the talon parts of vultures adjust the relative rotation of the middle phalanges of each toe to enable the sharp talons at the ends of each toe to clamp the target object, while the toes of the legs themselves do not come into contact with the target object. The above results show that the way vultures capture target objects is closely related to the physical information such as the phalangeal chain structure of their legs, the distribution characteristics and functions of each toe, and the proportion of each phalanx. Therefore, in order to achieve an ideal bionic effect, the design of the bio-inspired phalangeal chain gripper needs to take these factors into consideration.

### Extraction of critical biomimetic elements from vulture legs

Building upon the analytical results presented in the preceding section, cherry tomatoes were selected as the target for grasping. In alignment with the design requirements of a bionic end-effector for harvesting cherry tomatoes, mechanical biomimetics and information and control biomimetic methodologies were employed to extract physiological structural characteristics, motion dynamics, and essential biomimetic elements of vulture’s legs during prey capture. This approach provides valuable insights into the development of a bio-inspired phalangeal chain gripper specifically designed for picking cherry tomatoes. The key biomimetic elements are outlined as follows:

Firstly, the sharp talons are simplified or omitted to mitigate potential fruit damage. Secondly, the phalangeal chain of each toe is connected in series to form a multi-link mechanism. The links are connected by revolute joints, and a tendon-driven mechanism is adopted to achieve coordinated movement between the toes. Then, the number and length of each phalange vary, and each toe has a different function. Therefore, it is necessary to simplify and choose some toes, so as to select the appropriate number and length of phalanges, and finally determine the length and number of connecting rods. Next, the nervous system is simplified as a pressure sensor for sensing a captured object. Materials with high friction coefficient are used as the epidermis and elastic muscle to reduce the mechanical damage to the target fruit. Finally, in terms of the capturing pattern of the target object, the target is clamped by sharp talons at the end of each toe or by the leg’s toe envelope.

### Structural design of bio-inspired phalangeal chain gripper

**Fig 2:**
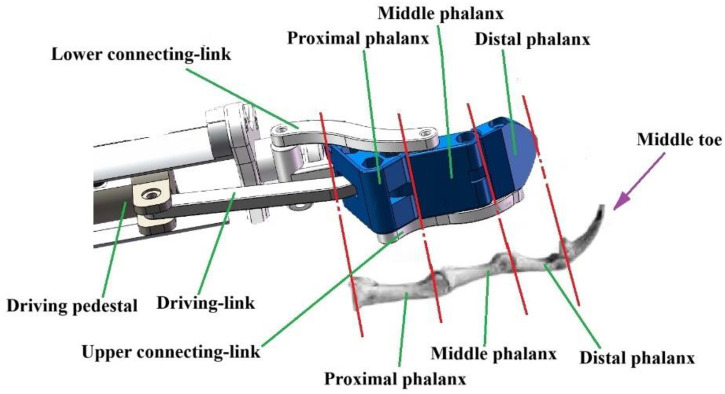
Structure of the bio-inspired Phalangeal chain gripper inspired by the phalangeal chain of the middle toe.

A bio-inspired phalangeal chain gripper, modeled after the phalangeal chain in vulture’s legs, was designed to handle smooth, spherical cherry tomatoes (Fig 2). While vultures’ strong talons enable them to grasp prey with precision, replicating this feature risks damaging the fruit during harvesting. To prevent this, the talon-like elements were omitted. The gripper, mimicking the segmented structure of a vulture’s phalangeal chain, consists of three phalanges of varying lengths—distal, middle, and proximal—connected by motors. This design ensures flexibility but introduces challenges. Adding motors between phalanges increases mass and volume, hindering performance in complex environments and raising the likelihood of picking failure. Additionally, controlling multiple motors simultaneously complicates operation, potentially reducing harvesting efficiency. Despite advancements in underdrive technology, these issues remain [29,30]. To address these issues, the gripper was designed as an active system with segmented features, similar to the controllable phalangeal chain of a vulture’s middle toe. The design maintains functionality while balancing structural and operational demands. Due to the coordinated movement of the phalangeal chain of the vulture’s toes being jointly achieved by tendons and rotations between the phalanges, the connection between the three phalanges in the bio-inspired phalangeal chain gripper is replaced with a hinge in the form of a rotational pair. The coordinated movement of the three phalanges is facilitated by an underactuated mechanism, which is mainly composed of a driving pedestal, an upper connecting-link, a lower connecting-link, and a driving-link, thereby achieving coordinated movement between the toes of the bio-inspired phalangeal chain gripper.

### Structural design of bionic end-effector

**Fig 3:**
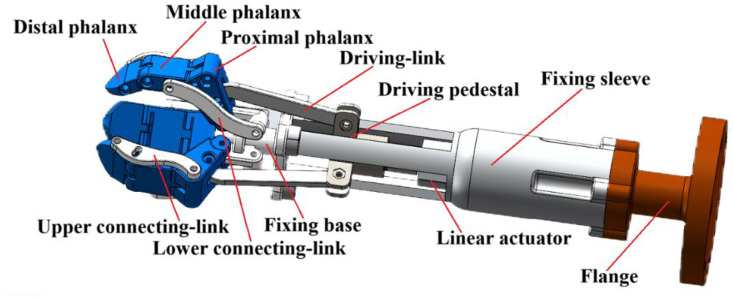
The structure of the bionic end-effector.

A bionic end-effector, integrating a bio-inspired phalangeal chain gripper and typical picking patterns, was developed for efficient fruit harvesting across various orientations. The end-effector comprises a flange, fixing sleeve, linear actuator, driving pedestal with three lugs, fixing base, and three bio-inspired phalangeal chain grippers (Fig 3). The bio-inspired phalangeal chain gripper is mainly composed of a distal phalanx, an upper connecting-link, a middle phalanx, a lower connecting-link, a proximal phalanx, and a driving-link. The flange, fixing sleeve, driving pedestal, and three bio-inspired phalangeal chain grippers are 3D-printed from resin. The flange is bolted to a 7-DOF space manipulator on one end and the fixing sleeve on the other. A linear actuator is housed within the fixing sleeve, with its pushrod connected to the driving pedestal. The fixing base connects three bio-inspired phalangeal chain grippers on one side and the fixing sleeve on the other. The driving-link attaches to the proximal phalanx and the driving pedestal. The upper connecting-link connects the distal phalanx’s midpoint to the upper section of the proximal phalanx. Similarly, one end of the lower connecting-link is positioned in the middle of the distal phalanx, and its other end is secured in the middle of the fixing base. The distal phalanx, middle phalanx, and proximal phalanx are sequentially interconnected. These components are fastened together using bolts. When the linear actuator is powered and the target fruit is positioned between the three middle phalanges, the actuator moves the driving pedestal forward. This, in turn, pushes the connecting rod, causing the three proximal phalanges to move inward. Constrained by the upper and lower connecting-links, the middle and distal phalanges of each bio-inspired gripper moved in opposite directions to the proximal phalanges. The three grippers close in on the fruit until the actuator’s pushrod completed its stroke, successfully capturing the target fruit. This progressive, multi-point closing mode enables the end-effector to accommodate fruit-size variation and positioning error more effectively than a single-stage squeeze, while also distributing the contact load more evenly to reduce local damage and fruit slippage.

### Main instruments and equipment

**Fig 4:**
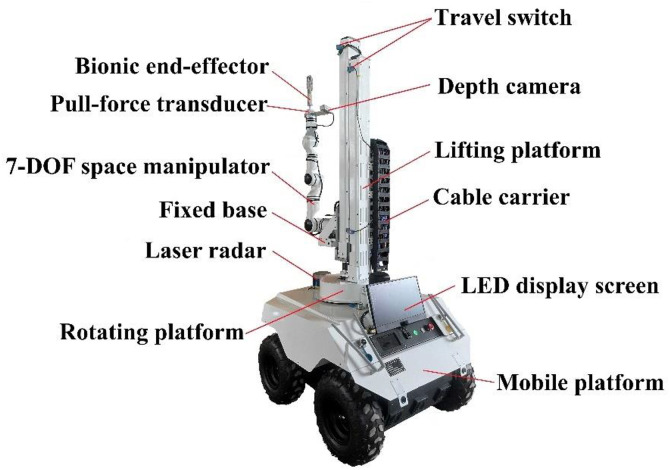
Harvesting robot for cherry tomatoes.

The cherry tomato picking experiment used an intelligent picking robot (Fig 4), comprising a bionic end-effector, a 7-DOF space manipulator (RM-75B, REALMAN, Beijing), a rotating platform, a lifting platform, a control platform, and a mobile platform (Bulldog, SUNSPEED, Shandong). The lifting platform includes a cable carrier, three travel switches, a fixed seat, and two vertically installed ballscrew-type electric actuators. The bionic end-effector is mounted at the end of the 7-DOF manipulator, which is fixed to the side of the lifting platform. The lifting platform is attached to the rotating platform, which is secured on the mobile platform. The 7-DOF manipulator, factory-calibrated, has a 610 mm working radius, ± 0.05 mm positioning accuracy, and weighs 7.8 kg. The mobile platform, also factory-calibrated, offers a top speed of 2 ms^-1^, a 100 kg load capacity, ± 10 mm positioning accuracy, a 0 m turning radius, and a maximum climbing angle of 40°. The bionic end-effector at the 7-DOF manipulator’s end harvests fruit following the pre-set picking pattern.

### Experimental method of cherry tomato harvesting

A total of 29 robotic cherry tomato picking tests were conducted. The robot first mapped the experimental site and located itself relative to the target plant using a laser inertial system. A binocular depth camera on the 7-DOF manipulator captured images, and the image recognition system calculated the 3D coordinates of each fruit, transmitting ripe fruit data to the robot’s upper computer. The upper computer then relayed the coordinates to the lower computer, which issued instructions to the rotating platform, lifting platform, 7-DOF manipulator, and bionic end-effector. The rotating platform adjusted to align the manipulator with the target fruit, the lifting platform moved to the required height, and the manipulator and end-effector worked together to harvest the fruit using a predefined pattern. Specifically, the manipulator aligned with the target fruit, and the linear actuator closed the phalangeal grippers to secure it. The end-effector then retracted at 3.0 mm·s ⁻ ¹ until the fruit detached. The harvested fruit was subsequently placed in a circular container before the manipulator proceeded to the next position. These 29 runs refer to the structural-parameter measurement experiments used for the RSM-BBD optimization (Table 2), rather than the later harvesting performance validation tests.

During the experiment, key parameters were kept constant: the end-effector’s retraction speed (3 mm·s⁻¹), retraction distance (30 mm), and the linear actuator’s speed (15 mm·s⁻¹). The retraction speed of 3 mm·s⁻¹ ensured the end-effector’s performance was tested under quasi-static conditions. Measurements of the end-effector’s dimensions (diameter, length, and distance in open or clamping states) were recorded with a vernier caliper (0.01 mm precision) before and after each attempt.

### Response surface design

**Fig 5:**
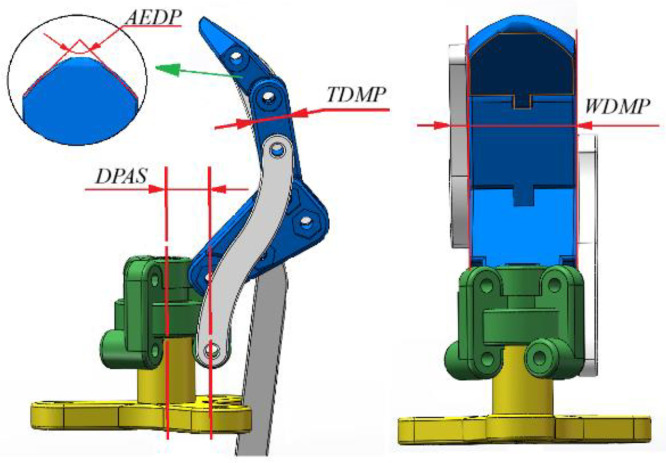
The schematic illustration of the four independent variables on the bionic end-effector.

A randomized response surface methodology, widely used in agricultural optimization studies, was employed [31–33]. From previous single-factor tests, four key variables influencing the bionic end-effector’s performance were identified and are defined here at their first mention: X_1_ denotes the width of the distal phalanges and middle phalanges (WDMP, mm), X_2_ denotes the thickness of the distal phalanges and middle phalanges (TDMP, mm), X_3_ denotes the angle at the end of the distal phalanx (AEDP, °), and X_4_ denotes the distance between the heel phalanges and the central axis of the fixed seat (DPAS, mm). Each independent variable is shown in Fig 5, and its range of values is listed in Table 1 (Fig 5 and Table 1). Fruit size and mass were also not parameterized as Box-Behnken factors, because the optimization was designed to isolate the structural contribution of the end-effector while using fruit samples from a controlled maturity stage and a narrow size range. To isolate the structural contribution of the end-effector in the RSM-BBD optimization, environmental factors such as fruit orientation, local plant density, and pedicel stiffness were not parameterized as Box-Behnken factors at this stage, but were treated as external harvesting conditions for later validation.

**Table 1:** Factors and levels of the Box-Behnken design.

Symbols	Input variables	units	Levels		
			Low (−1)	Medium (0)	High (+1)
*X*_1_	WDMP	mm	12	16	20
*X*_2_	TDMP	mm	4	5	6
*X*_3_	AEDP	°	75	115	155
*X*_4_	DPAS	mm	6	7	8

During the parameter measurement experiments for the bionic end-effector, the maximum inscribed circle diameter of the middle phalanges (*R*_1_, DCMP) was measured in the gripping state using a vernier caliper. Similarly, the maximum inscribed circle diameter of the distal phalanges (*R*_2_, DCDP) was recorded during fruit picking. To ensure accuracy, the average of ten measurements was calculated for both DCMP and DCDP. For each BBD run, both response variables were measured 10 times under the same parameter setting, and the average values were used for the subsequent regression and ANOVA.

Larger DCMP values reduce the risk of cherry tomatoes being squeezed and damaged, but excessive sizes may compromise the gripper’s flexibility. Conversely, small DCMP values can cause damage to the fruit, impacting later storage, transport, or processing. For DCDP, a smaller value limits the ability to handle larger fruits, while larger values may allow stems, stalks, or leaves to interfere with gripping, lowering harvesting success. Therefore, DCMP and DCDP were treated as structural surrogate indices for fruit damage risk and harvesting stability, respectively. Considering the measured equatorial diameter of the cherry tomatoes (25.88 mm ± 1.75 mm) and repeated grasping observations, the target values were set to DCMP = 27 mm and DCDP = 41 mm to jointly balance low compression damage and stable fruit retention. Based on these considerations, optimal values were determined as DCMP = 27 mm and DCDP = 41 mm.

Using Design-Expert 6.0.8 software, a Box-Behnken Design matrix was created for four factors at three levels, as shown in Table 1, with DCMP(R_1_) and DCDP(R_2_) as response variables. A total of 29 experiments were conducted using RSM-BBD to model the relationships between key parameters and end-effector performance, with the average results summarized in Table 2. ANOVA validated the regression model and analyzed the quadratic effects of key factors on the response function (Tables 3 and 4). The optimized parameter combination was subsequently validated through harvesting experiments using harvesting success rate, plant damage, and post-harvest fruit quality variation as practical performance indicators.

**Table 2:** Experimental design with four independent variables at three levels and corresponding response simulation results.

Run	Parameters				Output response	
	*X*_1_	*X*_2_	*X*_3_	*X*_4_	DCMP(*R*_1_)	DCDP(*R*_2_)
1	12	5	115	8	28.5	42.5
2	20	5	115	8	28	41.5
3	16	4	115	8	30	44
4	16	5	115	7	27	40.5
5	12	6	115	7	25	37
6	16	5	115	7	27	40.5
7	16	4	115	6	26.5	39
8	16	5	155	6	24.5	37.5
9	20	5	75	7	26	39.5
10	16	6	115	8	26.5	40.5
11	16	5	75	6	24	38.5
12	16	6	115	6	23	37
13	16	5	75	8	28	43
14	16	5	155	8	27.5	42.5
15	20	5	115	6	24	36.5
16	16	5	115	7	27	40.5
17	20	5	155	7	26	38.5
18	12	4	115	7	28	40.5
19	16	4	155	7	28.5	41.5
20	16	6	75	7	25	39
21	12	5	115	6	24	37
22	16	4	75	7	28.5	43
23	12	5	155	7	28	39
24	16	6	155	7	27.5	38.5
25	20	6	115	7	26	36.5
26	12	5	75	7	26.5	40
27	16	5	115	7	27	40.5
28	20	4	115	7	28	42
29	16	5	115	7	27	40.5

**Table 3:** Variance analysis of BBD for DCMP.

Items	Sum of Squares	DOF	Mean Square	*F*-Value	*P*-value
Model *X*_1_ *X*_2_ *X*_3_ *X*_4_ *X*_1_*X*_2_ *X*_1_*X*_3_ *X*_1_*X*_4_ *X*_2_*X*_3_ *X*_2_*X*_4_ *X*_3_*X*_4_ *X*_1_^2^ *X*_2_^2^ *X*_3_^2^ *X*_4_^2^ Residual Lack of Fit Pure Error Cor Total	74.30 0.021 1.09 2.76 10.55 0.25 0.56 0.063 1.56 0.000 0.25 0.63 0.41 0.025 3.65 4.15 4.15 0.000 78.45	14 1 1 1 1 1 1 1 1 1 1 1 1 1 1 14 10 4 28	5.31 0.021 1.09 2.76 10.55 0.25 0.56 0.063 1.56 0.000 0.25 0.63 0.41 0.025 3.65 0.30 0.41 0.000	17.92 0.070 3.69 9.30 35.62 0.84 1.90 0.21 5.28 0.000 0.84 2.14 1.37 0.086 12.32	< 0.0001 0.7947 0.0752 0.0086 < 0.0001 0.3738 0.1898 0.6530 0.0376 1.0000 0.3738 0.1657 0.2615 0.7742 0.0035
Model Summary Statistics					
Std. Dev.	0.54		*R*-Squared	0.9472	
Mean	26.64		Adj. *R*-Squared	0.8943	
C.V.%	2.04		Pred. *R*-Squared	0.6956	
PRESS	23.88		Adeq Precision	16.608	

^a^Note: (1) *R*^2^ = 0.9472, Adj. *R*^2^ = 0.8943; (2) * and ** represent significant difference at *P* ＜ 0.05 and *P* ＜ 0.01, respectively.

**Table 4:** Variance analysis of BBD for DCDP.

Items	Sum of Squares	DOF	Mean Square	*F*-Value	*P*-value
Model *X*_1_ *X*_2_ *X*_3_ *X*_4_ *X*_1_*X*_2_ *X*_1_*X*_3_ *X*_1_*X*_4_ *X*_2_*X*_3_ *X*_2_*X*_4_ *X*_3_*X*_4_ *X*_1_^2^ *X*_2_^2^ *X*_3_^2^ *X*_4_^2^ Residual Lack of Fit Pure Error Cor Total	120.90 1.17 6.97 0.13 12.00 1.00 0.000 0.063 0.25 0.56 0.063 9.47 0.28 0.011 0.045 3.29 3.29 0.000 124.19	14 1 1 1 1 1 1 1 1 1 1 1 1 1 1 14 10 4 28	8.64 1.17 6.97 0.13 12.00 1.00 0.000 0.063 0.25 0.56 0.063 9.47 0.28 0.011 0.045 0.24 0.33 0.000	36.73 4.98 29.63 0.55 51.04 4.25 0.000 0.27 1.06 2.39 0.27 40.28 1.20 0.048 0.19	< 0.0001 0.0424 < 0.0001 0.4691 < 0.0001 0.0582 1.0000 0.6142 0.3200 0.1442 0.6142 < 0.0001 0.2923 0.8299 0.6683
Model Summary Statistics					
Std. Dev.	0.48		*R*-Squared	0.9735	
Mean	39.90		Adj. *R*-Squared	0.9470	
C.V.%	1.22		Pred. *R*-Squared	0.8473	
PRESS	18.96		Adeq Precision	23.896	

^a^ Note: (1) *R*^2^ = 0.9735, Adj. *R*^2^ = 0.9470; (2) * and ** represent significant difference at *P* ＜ 0.05 and *P* ＜ 0.01, respectively.

Design-Expert 6.0.8 was selected because it provides a mature implementation of Box-Behnken design generation, quadratic response-surface fitting, ANOVA, and desirability-based multi-response optimization, which together match the statistical workflow of the present study. A quadratic response-surface model based on BBD was chosen because the study involved four structural factors at three levels and aimed to estimate curvature and two-factor interactions efficiently during structural optimization. Compared with a full factorial design, BBD substantially reduces the number of required experimental runs while remaining suitable for estimating second-order effects.

The 29-run design was generated in randomized order in Design-Expert and included five replicated center points (Runs 4, 6, 16, 27, and 29 in Table 2) to estimate pure error and evaluate lack of fit.

## Results and discussion

### Variance analysis

Tables 3 and 4 present the ANOVA results for DCMP and DCDP, respectively. A *P*-value < 0.05 at a 95% confidence level indicates model significance, and the large F-values further support the adequacy of the quadratic models. For DCMP, *R*² was 0.9472 and Adj. *R*² was 0.8943, indicating that the model explained most of the observed variance. However, the Pred. *R*² was 0.6956, which lower than the Adj. *R*² and therefore suggests weaker out-of-sample predictive ability for this response than for DCDP. For DCDP, *R*², Adj. *R*², and Pred. *R*² were 0.9735, 0.9470, and 0.8473, respectively, indicating better predictive stability within the studied factor space. Adequate Precision values of 16.608 and 23.896 for DCMP and DCDP, respectively and thus indicated sufficient signal for optimization.

**Fig 6:**
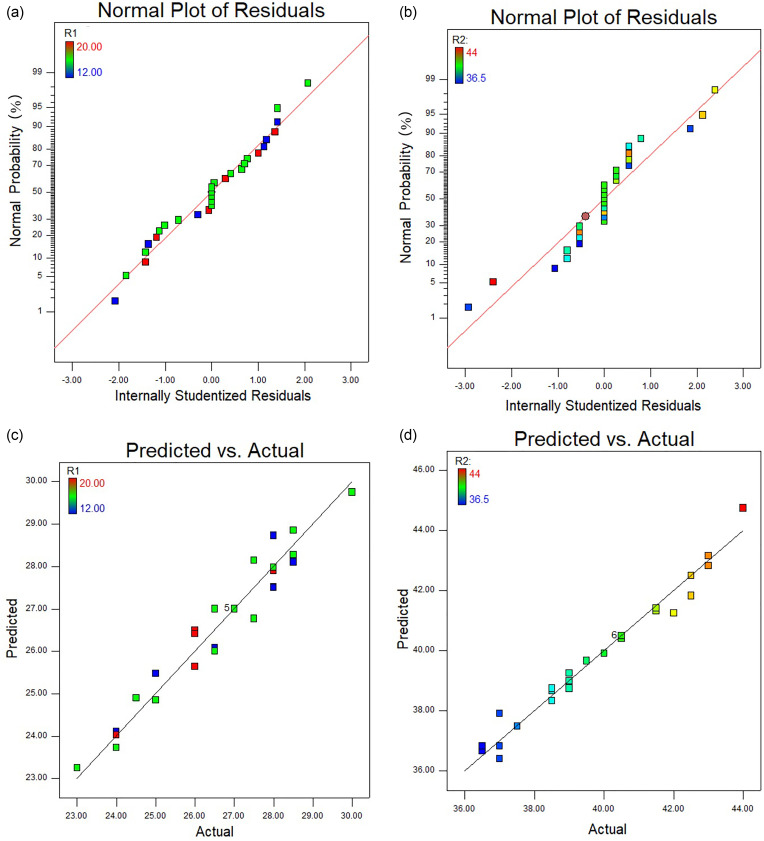
(a) Residuals plot of DCMP. (b) Residuals plot of DCDP.

Normal residual plots validate the effectiveness of the models (Fig 6a and 6b). Fig 6c demonstrates a linear fit between actual and predicted values, confirming the model’s high accuracy in predicting DCMP. Similarly, Fig 6d shows comparable results for DCDP. The final mathematical equations for DCMP(*R*_1_) and DCDP(*R*_2_) are presented in Eq. (1) and Eq. (2), respectively.

To supplement the visual residual analysis, statistical tests were carried out on the model residuals. For DCMP, the Shapiro-Wilk test yielded p = 0.431, indicating no significant departure from normality. For DCDP, the Shapiro-Wilk test yielded p = 0.013, suggesting mild non-normality; therefore, this model was interpreted with additional caution and judged jointly with the residual trend, ANOVA significance, and confirmatory experiments rather than on normality alone. In addition, because the five replicated center points produced identical averaged responses, the lack-of-fit partition provided limited extra discrimination in this dataset. Therefore, independent confirmatory experiments were emphasized to verify the predictive usefulness of the fitted equations.


$$\begin{array}{c} \begin{array}{c} R_{\text{1}}=\;-14.41764+0.75911X_{\text{1}}-6.67187X_{\text{2}}+0.020443X_{\text{3}}+13.59375X_{\text{4}}\\ +0.062500\;X_{\text{1}}X_{\text{2}}-\left(2.34375E-003\right)X_{\text{1}}X_{\text{3}}-0.031250X_{\text{1}}X_{\text{4}}\\ +0.015625X_{\text{2}}X_{\text{3}}-\left(5.21805E-015\right)X_{\text{2}}X_{\text{4}}-\left(6.25000E-003\right)X_{\text{3}}X_{\text{4}}\\ -0.019531{X_{\text{1}}}^{\text{2}}+0.25000{X_{\text{2}}}^{\text{2}}-\left(3.90625E-005\right){X_{\text{3}}}^{\text{2}}-0.75000{X_{\text{4}}}^{\text{2}} \end{array} \end{array}$$ (1)

$$\begin{array}{c} \begin{array}{c} R_{\text{2}}=-14.14518+3.22917X_{\text{1}}+4.19792X_{\text{2}}-0.070573X_{\text{3}}+5.55729X_{\text{4}}\\ -0.12500X_{\text{1}}X_{\text{2}}+\left(1.70003E-017\right)X_{\text{1}}X_{\text{3}}-0.031250X_{\text{1}}X_{\text{4}}\\ +\left(6.25000E-003\right)X_{\text{2}}X_{\text{3}}-0.37500X_{\text{2}}X_{\text{4}}+\left(3.125000E-003\right)X_{\text{3}}X_{\text{4}}\\ -0.075521{X_{\text{1}}}^{\text{2}}-0.20833{X_{\text{2}}}^{\text{2}}+\left(2.60417E-005\right){X_{\text{3}}}^{\text{2}}-0.083333{X_{\text{4}}}^{\text{2}} \end{array} \end{array}$$ (2)

**Fig 7:**
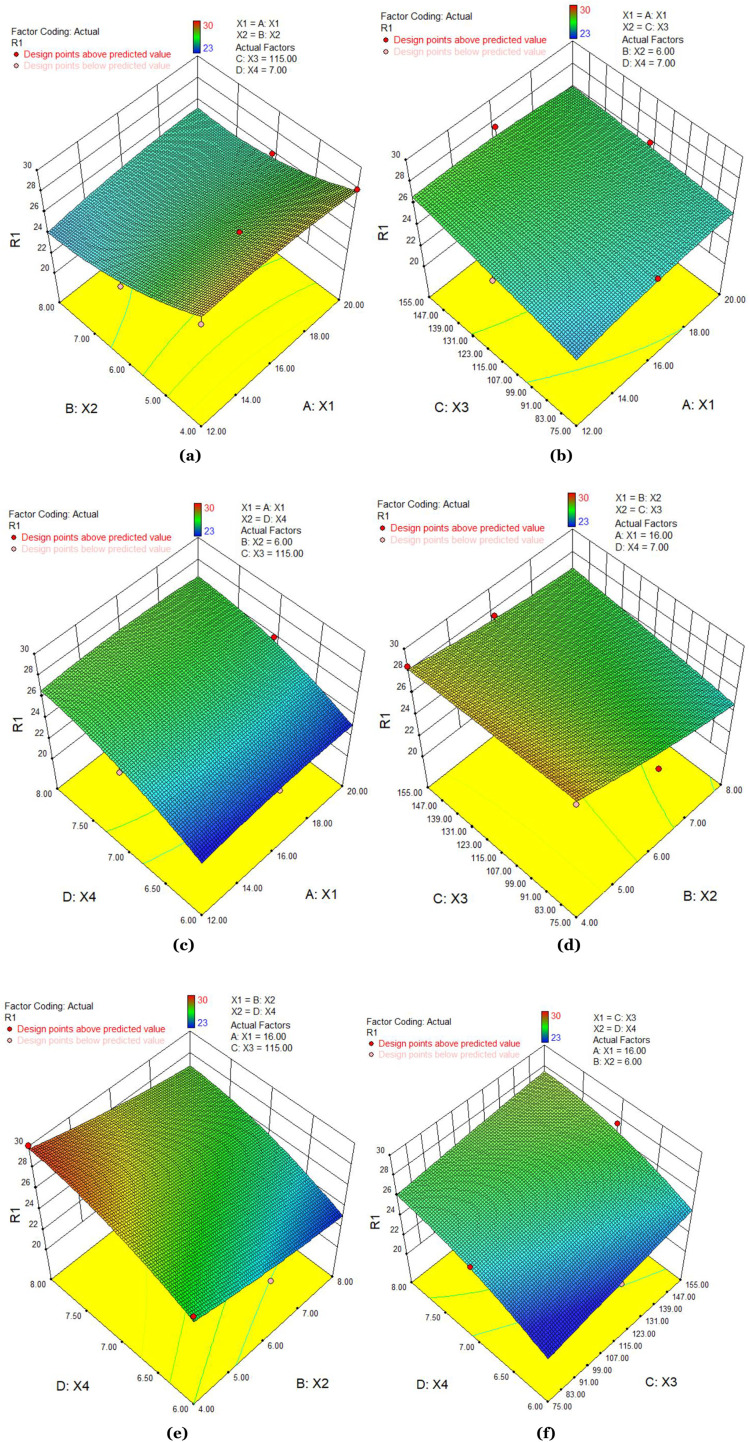
Interaction plots of DCMP. (a) Interaction plots for the interactive effects of WDMP, and TDMP on DCMP; (b) Interaction plots for the interactive effects of WDMP, and AEDP on DCMP; (c) Interaction plots for the interactive effects of WDMP, and DPAS on DCMP; (d) Interaction plots for the interactive effects of TDMP, and AEDP on DCMP; (e) Interaction plots for the interactive effects of TDMP, and DPAS on DCMP; (f) Interaction plots for the interactive effects of AEDP, and DPAS on DCMP.

**Fig 8:**
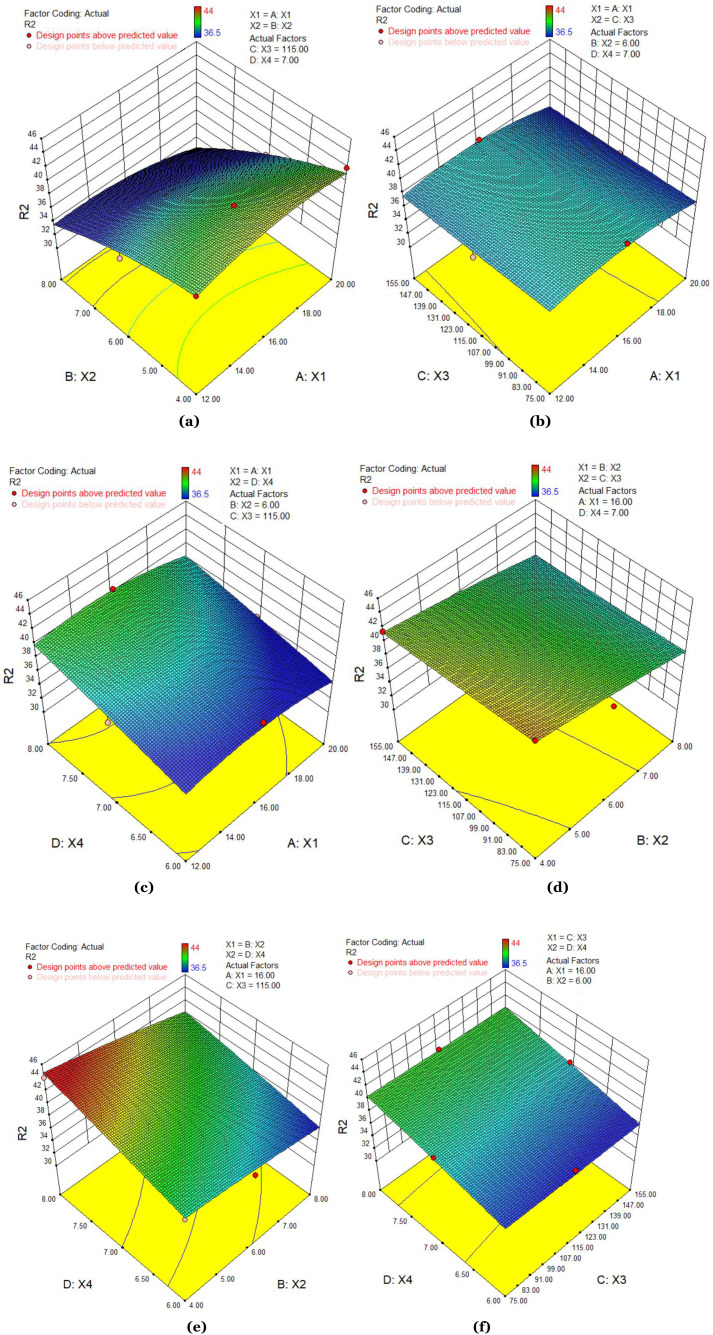
Interaction plots of DCDP. (a) Interaction plots for the interactive effects of TDMP, and DPAS on DCDP; (b) Interaction plots for the interactive effects of WDMP, and AEDP on DCDP; (c) Interaction plots for the interactive effects of WDMP, and DPAS on DCDP; (d) Interaction plots for the interactive effects of TDMP, and AEDP on DCDP; (e) Interaction plots for the interactive effects of WDMP, and TDMP on DCDP; (f) Interaction plots for the interactive effects of AEDP, and DPAS on DCDP.

To visualize the interactive effects of *X*_1_, *X*_2_, *X*_3_, and *X*_4_ on DCMP and DCDP, 3D response surfaces and 2D contour plots are shown in Fig 7 and Fig 8, respectively. Table 3 indicates that three parameters (*X*_2_, *X*_3_, and *X*_4_) and four interactions (*X*_2_*X*_3_, *X*_1_*X*_3_, *X*_1_*X*_2_, and *X*_3_*X*_4_) significantly affect DCMP, with *X*_4_ being the most influential, as reflected in its F-value. Fig 7 shows that DCMP increases with *X*_3_ and *X*_4_ but decreases rapidly with *X*_2_. Changes in *X*_1_ have minimal impact, with DCMP initially rising slightly as *X*_1_ increases before declining gradually after reaching a peak.

As depicted in Fig 7(c, e, f), increasing *X*_4_ from 6 mm to 8 mm raises DCMP from 23 mm to 30 mm. This occurs because greater *X*_4_ values increase the distance between each phalange and the bio-inspired phalangeal chain gripper’s central axis, expanding the space around the fruit and enlarging DCMP. However, excessive distance enlarges the bio-inspired phalangeal chain gripper, allowing fruit stems, stalks, or leaves to obstruct the holding space, hindering successful grasping and reducing the picking success rate.

From Fig 7(a, c, e), it can also be seen that as *X*_2_ increases from 4 mm to 6 mm, DCMP decreases from 30 mm to 23 mm. This was primarily because of the fact that *X*_2_ increases the thickness of the middle and distal phalanges, thereby reducing the inner envelope space for the fruit in the bionic end-effector, and ultimately resulting in a reduced DCMP. Conversely, excessive *X*_2_ will reduce the strength and stiffness of the phalanges in the biomimetic grippers, the middle and distal phalanges are susceptible to deformation, and the risk of fruit mechanical damage increases.

From Fig 7(b, d, f), *X*_3_ and *X*_4_ have similar effects on DCMP, but too small *X*_3_ will sharpen the end of the distal phalanges. When the biomimetic grippers are in the open state, the end of the distal phalanges are easy to puncture the fruit, resulting in unrecoverable mechanical damage to the fruit. On the contrary, too large *X*_3_ will increase DCMP, but it will affect the flexibility of the biomimetic grippers in the clamping state, which is more unfavorable for the path planning of the 7-DOF spatial manipulator in the unstructured picking environment.

Fig 7(a-c) shows that DCMP reaches a maximum of 30 mm at high *X*_1_ values. As *X*_1_ increases from 16 mm to 20 mm, DCMP gradually decreases. While *X*_1_ has minimal direct influence on DCMP, its interactions with *X*_2_, *X*_3_, and *X*_4_ significantly impact DCMP. Limiting specific parameters is essential to enhance the flexibility of the bio-inspired phalangeal chain gripper.

The contour curvatures in Fig 8 follow this order: Fig 7d> Fig 7b> Fig 7a = Fig 7f> Fig 7c> Fig 7e, indicating that the *X*_2_ and *X*_3_ interaction has the greatest effect on DCMP, followed by *X*_1_ and *X*_3_, *X*_1_ and *X*_2_, *X*_3_ and *X*_4_, and lastly *X*_2_ and *X*_4_. Interactions between *X*_1_ and *X*_3_, *X*_3_ and *X*_4_ have similar impacts, as disclosed in Table 3.

Table 4 highlights that *X*_1_, *X*_2_, and *X*_4_, along with five interactions (*X*_1_*X*_2_, *X*_2_*X*_4_, *X*_2_*X*_3_, *X*_1_*X*_4_, and *X*_3_*X*_4_), significantly affect DCDP, with *X*_4_ having the greatest influence, as indicated by its high F-value. DCDP initially increases with *X*_1_ before gradually decreasing after reaching its peak. In contrast, DCDP decreases as *X*_2_ and *X*_3_ increase but rises rapidly with *X*_4_. This behavior differs from the parameter effects observed for DCMP.

As illustrated in Fig 8(c, e, f), increasing *X*_4_ from 6 mm to 8 mm raises DCDP from 36.5 mm to 44 mm. The reasons for this result are the same as the impact of *X*_4_ on DCMP, which will not be elaborated here. Similar to the effect of *X*_4_ on DCMP, when *X*_4_ increases, the thickness of the distal phalanges and middle phalanges increases. When the bio-inspired phalangeal chain grippers are in the clamping state, the space for the bio-inspired phalangeal chain grippers to envelope the fruit is reduced, resulting in DCDP becoming smaller.

Fig 8(a, d, e) shows that increasing *X*_2_ from 4 mm to 6 mm reduces DCDP from 42 mm to 38.5 mm. However, excessive *X*_2_ will lead to an increase in the thickness of both the middle and distal phalanges, consequently diminishing the space of the inner envelope fruit of the bionic end-effector, and ultimately resulting in a decreased DCDP. Additionally, although the influence rule of *X*_3_ on DCDP is similar to that of *X*_2_, the influence rule of *X*_3_ on DCDP is not significant, which is consistent with the result disclosed in Fig 8(d).

Fig 8(d) shows that DCDP initially increases with *X*_1_, reaches a peak, and then decreases as *X*_1_ continues to rise. While *X*_1_ has minimal direct influence on DCDP, its interactions with *X*_2_, *X*_3_, and *X*_4_ significantly affect DCDP, as confirmed by Table 4 and Fig 8(a-c). To maintain appropriate DCDP values, key gripper parameters must be controlled.

The contour line curvatures in Fig 8 follow this order: Fig 8a> Fig 8e> Fig 8d> Fig 8c = Fig 8f> Fig 8b. This indicates that the *X*_1_ and *X*_2_ interaction has the strongest effect on DCDP, followed by *X*_2_ and *X*_4_, *X*_2_ and *X*_3_, *X*_1_ and *X*_4_, *X*_3_ and *X*_4_, and finally *X*_1_ and *X*_3_ with the least significance. Similar to Table 3, interactions between *X*_1_ and *X*_4_, *X*_3_ and *X*_4_ have equivalent effects, as shown in Table 4.

Overall, these findings are consistent with the results presented in Table 3 (the *P*-values of *X*_2_*X*_3_, *X*_1_*X*_3_, *X*_1_*X*_2_, and *X*_3_*X*_4_ were 0.0376, 0.1898, 0.3738, and 0.3738, respectively) and Table 4 (the *P*-values of *X*_1_*X*_2_, *X*_2_*X*_4_, and *X*_2_*X*_3_ were 0.0582, 0.1442, and 0.3200, respectively). From a harvesting perspective, DCMP mainly reflects the compression tolerance of the grasping space, whereas DCDP mainly reflects the distal enclosure window for stable retention. For this reason, the parameter optimization in this study was formulated as a simultaneous multi-response problem.

### Optimization results

Design-Expert software was used for desirability analysis with target values of DCMP at 27 mm and DCDP at 41 mm. Input parameters (WDMP, TDMP, AEDP, and DPAS) and the resulting DCMP and DCDP are shown in Table 5.

**Table 5:** Key parameter constraints and responses.

Name	Lower Limit	Upper Limit	Importance
WDMP (mm)	12	20	3
TDMP (mm)	4	6	3
AEDP (°)	75	155	3
DPAS (mm)	6	8	3
DCMP (mm)	23	30	3
DCDP (mm)	36.5	44	3

The primary goal of this study was to identify optimal settings for achieving the desired outcomes. An RSM-BBD-based model predicted 25 optiomal desirability outcomes with target values of DCMP at 27 mm and DCDP at 41 mm, as shown in Table 6. Five optimal solutions from Table 6, none of which were part of the 29-run BBD fitting dataset, were selected as confirmatory tests to externally verify the response-surface equations. Table 7 summarizes the predicted RSM values, experimental test results, mean values, standard deviations, and prediction errors. All prediction errors were below 5%, and the mean absolute percentage errors were 2.73% for DCMP and 1.97% for DCDP, confirming that the fitted equations were sufficiently reliable for optimization within the investigated design space.

**Table 6:** Optimal combinations for multi-response desirability optimization.

Number	*X*_1_	*X*_2_	*X*_3_	*X*_4_	DCMP(mm)	DCDP(mm)	Desirability
1 2 3 4 5 6 7 8 9 10 11 12 13 14 15 16 17 18 19 20 21 22 23 24 25	16.39 16.38 14.75 16.67 16.88 15.12 13.35 16.60 13.48 17.89 12.76 15.63 17.07 15.66 17.50 17.44 12.83 14.09 15.81 15.43 18.13 17.89 18.09 16.04 18.35	4.94 5.30 5.78 4.13 4.98 4.64 5.16 5.62 5.43 4.26 5.55 4.53 4.38 4.94 4.92 4.69 4.82 5.60 4.72 5.09 5.30 5.00 4.24 4.53 5.54	91.90 93.01 104.80 79.07 89.63 87.13 89.91 94.97 96.97 77.28 100.05 86.68 84.01 92.99 84.42 84.92 76.19 101.44 90.40 94.36 75.13 79.44 75.04 87.56 78.18	7.06 7.40 7.95 6.41 7.11 6.82 7.40 7.82 7.64 6.52 7.88 6.72 6.60 7.05 7.08 6.87 7.17 7.77 6.86 7.19 7.56 7.17 6.51 6.71 7.97	27 27 27 27 27 27 27 27 27 27 27 27 27 27 27 27 27 27 27 27 27 27 27 27 27	41 41 41 41.0001 41 41 41 41.0001 41 41 40.9999 41 41 41 41 40.9999 41 41.0001 40.9999 41 41 40.9999 41.0001 40.9999 41	1 1 1 1 1 1 1 1 1 1 1 1 1 1 1 1 1 1 1 1 1 1 1 1 1

**Table 7:** Validation test results.

No.	*X*_1_	*X*_2_	*X*_3_	*X*_4_	DCMP (mm)	Prediction error (%)	DCDP (mm)	Prediction error (%)
					results		results	
1 2 3 4 5	16.38 13.35 15.66 15.43 18.35	5.30 5.16 4.94 5.09 5.54	93.01 89.91 92.99 94.36 78.18	7.40 7.40 7.05 7.19 7.97	26.85 28.14 26.51 27.90 28.03	0.6 4.2 1.8 3.3 3.8	41.73 41.31 41.64 42.87 41.49	0.76 1.78 1.55 4.55 1.19

The regression model for DCMP and DCDP identified the optimal conditions as X1 16.38 mm, X2 5.30 mm, X3 93.01°, and X4 7.40 mm (Tables 6 and 7). For practical simplicity, a verification experiment was conducted with adjusted parameters: X1 16 mm, X2 5 mm, X3 90°, and X4 7.5 mm. The experimental results for DCMP and DCDP were 27 mm ± 0.67 mm and 41 mm ± 0.55 mm, respectively, closely matching the predicted values (27 mm and 41 mm).

### Performance test

**Fig 9:**
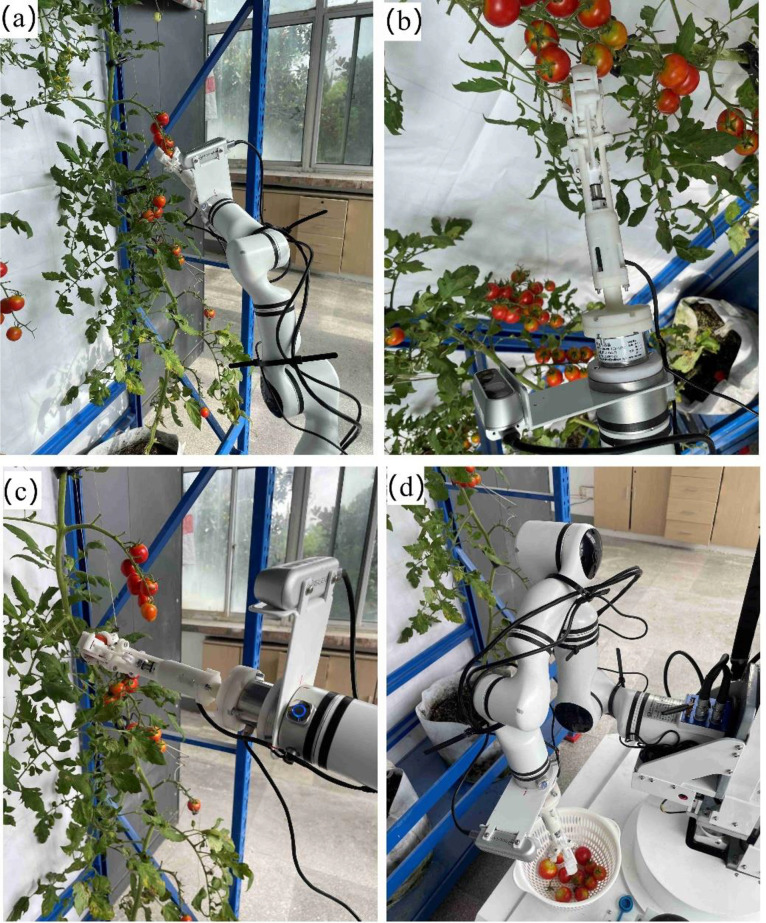
The picking process diagram of the optimized bionic end-effector (a) Close to the target fruit; (b) The target fruit will be grabbed; (c) The target fruit was successfully separated; (d) The target fruit is being dropped into a container.

To evaluate the performance of the optimized bionic end-effector, picking experiments were conducted in a plant factory. The harvesting process is illustrated in Fig 9 and detailed in Section “Experimental method of cherry tomato harvesting”. A total of 12 experiments were performed, sequentially numbered from 1 to 12. The first six groups followed the pulling picking pattern, while the last six adopted the integrated picking pattern, involving the end-effector stretching 40 mm and then twisting 180°. To ensure consistency with the pulling picking pattern, the Maximum Separation Force (MSF) direction between the fruit and plant was set opposite to the applied force in each experiment. For both patterns, the experiments were divided into six subgroups: maximum separation force, picking success rate, single-fruit picking time, plant damage, 72-hour fruit quality variability, and 168-hour fruit quality variability. Each small subgroup was tested 10 times, with each consecutive picking of 10 single fruits. To prevent random errors from introducing uncertainties to the experimental results, the positions of the cherry tomato plants within the cultivation layer were randomly altered and fruits at different height positions were selected for the picking experiments. Consequently, the validation stage exposed the optimized end-effector to naturally varying fruit positions, partial occlusion states, and local canopy density in the plant factory, although these environmental factors were not independently quantified in the response-surface model.

During the experiment, a force sensor installed between the 7-DOF manipulator and the bionic end-effector measured the maximum separation force. Post-experiment, data were recorded on the number of picked and unpicked fruits, plant damages, 72-hour and 168-hour quality changes, total single-fruit picking time, and individual picking time. Plant damage was defined as the occurrence of pedicel or floral axis breakage during harvesting. The total single fruit picking process time is the time required for the entire process from placing the current fruit in the circular container to before placing the next fruit in the circular container. The single fruit picking time only records the total time spent from identifying and picking a single fruit to placing it in the circular container, excluding the time for the rotation of the rotating platform and the adjustment of the lifting platform height by the lifting platform. Finally, the picking success rate, fruit damage rate, single-fruit picking time, and maximum separation force were statistically analyzed, with averages of all parameters and results recorded as the final outcomes. The results of fruit harvesting using different picking methods are presented in Tables 8 and 9.

**Table 8:** Results of cherry tomato picking experiment with pulling picking pattern.

No.	MSF(N)	Number of successful picking	single fruit picking process time (s)	single fruit picking time (s)	Number of plant damage	Number of 72-hour qualitative changes	Number of 168-hour qualitative changes
01	5.49	8	10.69	4.35	1	2	3
02	6.52	9	14.27	4.51	2	2	4
03	5.59	9	13.30	4.71	3	3	5
04	6.66	9	14.53	3.83	2	2	3
05	7.31	8	13.49	3.97	1	2	4
06	7.59	9	11.45	5.45	1	3	5
07	5.25	10	10.72	4.88	3	1	2
08	6.25	8	14.19	5.42	2	2	3
09	8.85	9	12.07	5.61	0	1	3
10	7.43	9	11.32	5.32	2	2	3

**Table 9:** Results of cherry tomato picking experiment with integrated picking pattern.

No.	MSF (N)	Number of successful picking	single fruit picking process time(s)	single fruit picking time(s)	Number of plant damage	Number of 72-hour qualitative changes	Number of 168-hour qualitative changes
01	1.96	9	13.99	6.61	0	2	3
02	2.63	10	15.06	6.21	1	1	3
03	1.68	9	14.44	5.88	0	0	1
04	1.92	9	13.30	5.08	0	1	2
05	1.87	9	13.83	6.59	1	1	2
06	1.21	8	14.48	5.51	0	2	2
07	2.63	9	11.31	6.35	0	1	3
08	1.94	10	15.96	6.48	1	0	1
09	1.41	9	15.48	6.46	0	2	3
10	2.86	9	15.79	5.62	0	1	2

As indicated in Table 8, among the 10 groups of pulling picking patterns, the number of successfully harvested fruits was 88, with an average success rate of 88%. The total time range of the single-fruit harvesting process was from 10.69 s to 14.53 s, with an average of 12.60 s ± 1.44 s. The single-fruit harvesting time range was from 3.83 s to 5.61 s, with an average of 4.81 s ± 0.61 s. The MSF range of harvested fruits was from 5.25 N to 8.85 N, with an average of 6.69 N ± 1.07 N. The number of plant damage incidents was 17, and the damage rate reached 17%. All the harvested fruits were left in the greenhouse for 3 days and 7 days. The number of fruits with texture changes was 20 and 35 respectively, and the fruit damage rates were 20% and 35% respectively. As presented in Table 9, within the 10 groups of integrated picking patterns, a total of 91 fruits were successfully harvested, with an average harvesting success rate of 91%. The total time range of the single-fruit harvesting process was from 11.31 s to 15.96 s, with an average of 14.36 s ± 1.31 s. The single-fruit harvesting time range was from 5.08 s to 6.61 s, with an average of 6.08 s ± 0.50 s. The MSF range of harvested fruits was from 1.21 N to 2.86 N, with an average of 2.01 N ± 0.51 N. The number of plant damage incidents was 3, and the damage rate was merely 3%. All the harvested fruits were left in the greenhouse for 3 days and 7 days. The number of fruits with texture changes was 11 and 22 respectively, and the fruit damage rates were 11% and 22% respectively. These field results provide practical support for the choice of DCMP and DCDP as optimization responses, because the optimized geometry simultaneously yielded high harvesting success and lower post-harvest fruit quality deterioration under the integrated picking pattern. However, a direct predictive regression between DCMP/DCDP and harvesting success rate or fruit damage for all 29 design combinations was not established in the present study and should be addressed in future work.

**Fig 10:**
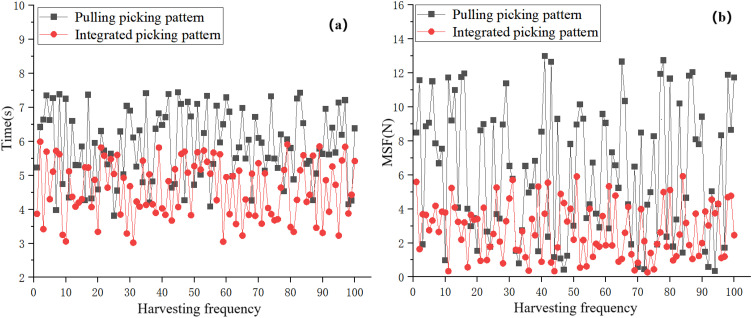
The single fruit picking time and MSF with picking frequency in different picking methods.

Based on the fruit-picking experiment outcomes of various picking patterns presented in Tables 8 and 9, the relationship curve between single fruit picking time and MSF with picking frequency is shown in Fig 10.

As depicted in Fig 10(a), in conjunction with the disclosed outcomes in Tables 8 and 9, in terms of single fruit picking time, the average total picking process duration per single fruit in the integrated picking pattern (14.36 s ± 1.31 s) exceeds that of the pulling picking pattern (12.60 s ± 1.44 s) by 1.76 s, while the average picking time per single fruit in the pulling picking pattern (3.83–5.61 s) is 1.27 s shorter than that in the integrated picking pattern (5.08–6.61 s). Despite the shorter single fruit picking time in the pulling picking pattern, compared with the operational efficiency of other cherry tomato harvesting robots, the single fruit picking time of the picking robot in this paper (6.08 s per fruit) is lower than that of other cherry tomato picking robots, which typically exceed 6.4 s per fruit [34–37].

Through the observation of fruit picking actions, it was discovered that the difference in picking time per single fruit between the two picking patterns is mainly manifested in the middle stage of fruit picking. In the initial and final stages of fruit picking, the bionic end-effector adopts the same initial and grasping-releasing postures, and the fruit picking paths of the bionic end-effector are similar, with the durations of the two picking patterns being essentially the same. In the middle stage of fruit picking, the picking action duration in the pulling picking pattern is 1.27 s, which is only 37.44% of that in the integrated picking pattern (approximately 2.03 s). The twisting action of the bionic end-effector in the integrated picking pattern is the reason for the increase in fruit picking time. The experiments also revealed that in a complete single fruit picking cycle, the times for the integrated picking action and the pulling picking action account for only 14.14% and 10.08% of the cycle time, respectively. Thus, it can be inferred that whether from the picking process or other process links, the movement speed and movement planning of the bionic end-effector are the keys to shortening the total time of the picking cycle.

In terms of the success rate of picking, the integrated picking pattern achieved a 91% success rate, 3.41% higher than the pulling pattern’s 88%. Additionally, the single-fruit harvesting success rate using the integrated picking pattern outperformed other tomato-harvesting robots [38–40]. Observations revealed that under identical operation scenarios, the bionic end-effector’s fault tolerance and the integrated picking pattern were crucial for improving the picking success rate. When the densely distributed cherry tomatoes were grasped by the bionic end-effector, the coordinate information position of the fruits changed, and the fault tolerance of the bionic end-effector could effectively reduce the adverse effects caused by the change in fruit position. When the visual positioning error was greater than 10.13 mm, the cherry tomato picking robot could still successfully complete the positioning, grasping, and picking with a relatively high harvesting success rate (91%). Furthermore, in the plant factory picking environment, the fruit’s color blended with the background, causing positioning deviations and preventing full enclosure of the fruits within the bionic end-effector’s clamping area. Using the pulling picking pattern often led to slippage between the fruits and the end-effector, increasing the risk of failure, particularly when the binding force between the fruits and plants was high. In contrast, the integrated picking pattern, with its twisting action, effectively disrupted the binding force. This allowed the end-effector to successfully separate fruits even when gripping only a partial area, achieving high harvesting success with reduced separation force.

In terms of MSF, the average MSF for the integrated picking pattern was only 30% of that of the pulling picking pattern. Observations showed that in the pulling pattern, the bionic end-effector gradually increased the separation force until it exceeded the fruit-plant binding force, successfully detaching the fruit. At this point, the average MSF reached 6.69 N ± 1.07 N. In contrast, the integrated picking pattern used a twisting motion followed by a fixed-direction pull. The twisting action disrupted the binding force, often separating the fruit from the plant. During the subsequent pulling phase, the required separation force was significantly lower, with an average MSF of 2.01 N ± 0.51 N. These results highlight that in the integrated picking pattern, the twisting motion primarily reduces the binding force, making separation easier. The end-effector applies only a portion of the total force needed for detachment, unlike the pulling pattern, where the end-effector provides the entire separation force.

As depicted in Fig 10(b), with respect to the MSF, the range of the MSF in the pulling picking pattern lies between 0.34 N and 12.99 N, and the range of the MSF in the integrated picking pattern is between 0.27 N and 5.92 N. Despite the existence of an overlap in the MSFs of the two picking patterns, the average MSF of the integrated picking pattern is smaller than that of the pulling picking pattern, suggesting that the integrated picking pattern is more capable of reducing the force exerted by the bionic end-effector. The plant thus experiences a smaller load and a lower risk of damage. Additionally, the individual differences in the binding force between the cherry tomato and the plant are the primary reason for the overlap in the MSFs of the two picking patterns.

In summary, compared with the pulling picking pattern, although the single fruit picking time of the cherry tomato picking robot using the integrated picking pattern increased by 26.40%, it still held an advantage over the single fruit picking time of similar picking robots. Moreover, the harvesting success rate increased by 2.22%, the MSF decreased by 69.96%, the plant damage rate decreased by 82.35%, the fruit damage rate within 72 hours decreased by 81.82%, and the fruit damage rate within 168 hours decreased by 59.09%. This suggests that the cherry tomato picking robot with the integrated picking pattern outperforms in harvesting success rate, MSF, fruit damage rate, and plant damage rate.

## Conclusion

This study designed a bionic end‑effector with three grippers, inspired by the mechanics of vulture legs, to reduce cherry tomato harvesting failures. The serial phalangeal-chain configuration provides progressive multi-point wrapping, which helps improve grasp stability under partial enclosure and mitigates concentrated squeezing during detachment. Key parameters influencing performance – WDMP, TDMP, AEDP, and DPAS – were optimized using the RSM-BBD method. The analysis identified DPAS and AEDP as the most significant factors for DCMP, while DPAS and TDMP were critical for DCDP, with WDMP and AEDP having minimal impact, respectively. The optimal conditions – WDMP of 16 mm, TDMP of 5 mm, AEDP of 90°, and DPAS of 7.5 mm – achieved the desired DCMP (27 mm) and DCDP (41 mm), with measured DCMP ranging from 23.0–30.0 mm and DCDP from 36.5–44.0 mm. In this study, DCMP and DCDP were used as surrogate indices of compression damage risk and grasping stability, respectively, and the harvesting tests of the optimized design (88% for the pulling pattern and 91% for the integrated picking pattern) provided practical validation for this multi-response optimization strategy. Experimental results validated the performance of the optimized end-effector, with a harvesting success rate of at least 88% within the tested fruit-size range. These harvesting results should therefore be interpreted as validation under the sampled fruit conditions rather than as evidence that fruit physical characteristics are universally negligible. Together with the lower separation force and lower plant damage and fruit damage observed in the integrated harvesting tests, these results confirm the intended improvement in mechanical stability, adaptability, and gentle picking performance. Notably, this research provides a reference for harvesting fruits with characteristics similar to cherry tomatoes, such as kumquats. Future work on integrating bionic end-effectors into cherry tomato harvesting could focus on selecting optimal picking patterns, improving image recognition accuracy, and enhancing localization technology. Nevertheless, the present study remains primarily a geometry-centered optimization. Future work should therefore couple structural parameters with biological and environmental variables such as fruit size, fruit mass, fruit orientation, local cluster density, and pedicel stiffness, in addition to improving image recognition accuracy, localization technology, and picking-pattern selection.
