# Supplementary material for: Optimization of a bionic end-effector for automated cherry tomato harvesting using Response Surface Methodology and Box-Behnken design
Source: PLoS One. 2026 Aug 14;21(8):e0352701. doi: 10.1371/journal.pone.0352701 (PMC13475895; doi:10.1371/journal.pone.0352701)
Supplement: Supplementary file 1 [file pone.0352701.s001.docx]

Dear academic editor and reviewers,

Re: Manuscript ID: PONE-D-25-68743 and Title: Optimization of a bionic end-effector for automated cherry tomato harvesting using response surface methodology and box-behnken design.

Thank you for your letter and the valuable comments from both the editor and the reviewers concerning our manuscript entitled "Optimization of a bionic end-effector for automated cherry tomato harvesting using response surface methodology and Box-Behnken design" (PONE-D-25-68743).

Overall, the comments have been fair, encouraging and constructive, and we have learned much from them. We sincerely appreciate the constructive suggestions from the academic editor, as well as the insightful feedback from the reviewers. All of these suggestions have been carefully considered.

After carefully studying the reviewers’ comments and your advice, we have made corresponding changes to the paper. Based on the instructions provided in your letter, we have uploaded the file of the revised manuscript. Appended to this letter is our point-by-point response to the comments raised by the reviewers. The comments are reproduced and our responses are given directly afterward in a different color. We would like also to thank you for allowing us to resubmit a revised copy of the manuscript.

We would like to thank you for allowing us to resubmit a revised copy of the manuscript and we highly appreciate your time and consideration.

If you have any question about this paper, please don’t hesitate to let me know.

Sincerely.

Dr Xie.

Response to the academic editor's comments:

We very much appreciate the careful reading of our manuscript and valuable suggestions of the academic editor. We have carefully considered the comments and have revised the manuscript accordingly. The comments can be summarized as follows:

**Q.** The manuscript addresses an important topic in agricultural robotics and presents a bio-inspired end-effector optimized using RSM and Box–Behnken Design. However, several methodological clarifications, stronger statistical validation, and improved explanation of experimental design and harvesting performance metrics are required before the manuscript can be considered for publication.

Response: Thank you for this constructive summary. We have revised the manuscript to address each of the three major concerns—methodological clarity, statistical rigor, and experimental design explanation—as detailed below.

To address the request for methodological clarifications, we have explicitly stated in the Introduction, in the section "Structural design of bio‑inspired phalangeal chain gripper", and in the section "Response surface design" the improvement logic of the present vulture‑inspired end‑effector over our previous design: mechanical performance is enhanced through coordinated under‑actuated motion of the proximal, middle, and distal phalanges; adaptability is improved by progressive self‑wrapping under fruit‑position deviation; and damage is reduced by removing talon‑like features and optimising contact geometry. Furthermore, in the Abstract, in the Introduction, and in the "Response surface design" section, we have clearly explained why DCMP (representing compressive clearance and thus squeezing damage risk) and DCDP (representing distal enclosure space and thus fruit retention stability) were selected as response variables, and why their target values (27 mm and 41 mm) were chosen as a compromise based on measured fruit size and repeated grasping observations.

To meet the requirement for stronger statistical validation, we have supplemented the replication and randomisation details in the sections "Response surface design" and "Experimental method of cherry tomato harvesting": the 29 BBD runs were generated in randomised order by Design‑Expert, included five replicated centre points, and each response was measured ten times per parameter combination to estimate pure error and lack of fit. In the "Variance analysis" section, we have added normality test results (Shapiro‑Wilk p = 0.431 for DCMP and p = 0.013 for DCDP), and we now interpret the DCDP model with additional caution, supported by ANOVA significance and confirmatory experiments. Moreover, in the optimisation results subsection (following the variance analysis) and in the newly added Table 7, we have included five independent confirmatory runs (not part of the original 29 BBD runs), all with prediction errors below 5%, confirming the predictive reliability of the response‑surface equations.

To address the need for a clearer explanation of the experimental design and harvesting performance metrics, we have explicitly distinguished, in the sections "Experimental method of cherry tomato harvesting" and "Response surface design", the 29 BBD runs for structural‑parameter optimisation from the later harvesting performance validation tests to avoid confusion. In the "Performance test" section, we note that the field‑validated success rates of the optimised end‑effector (88% for the pulling pattern and 91% for the integrated picking pattern) provide practical support for using DCMP and DCDP as optimisation responses, while acknowledging that a direct regression between these responses and success rate/damage metrics has not been established here and should be addressed in future work. Finally, in the "Performance test" section and in the "Experimental method of cherry tomato harvesting" section, we have provided clearer definitions of plant damage, single‑fruit picking time, and maximum separation force (MSF), and explained how the integrated picking pattern reduces MSF and plant damage compared to the pulling pattern. We believe these revisions have substantially improved the clarity, rigour, and reproducibility of the manuscript.

Response to the reviewer's comments:

We are very grateful to your comments for the manuscript. According to your advice, we amended the relevant part in manuscript. All of your questions were answered one by one.

Reviewer #1:

We very much appreciate the careful reading of our manuscript and valuable suggestions of the reviewer. We have carefully considered the comments and have revised the manuscript accordingly. The comments can be summarized as follows:

**Q1.** The authors mention earlier bionic grippers and previous success rates but do not clearly explain how the present design fundamentally improves mechanical performance, adaptability, or damage reduction.

Response: Thank you for this valuable suggestion. In response, we revised the manuscript in several locations to make the improvement logic explicit. First, in the Introduction, we now state that the limitations of our earlier cherry tomato gripper were mainly related to stable enclosure and damage control during fruit detachment. Second, we clarify that the present vulture-inspired design was conceived to improve mechanical performance through coordinated under-actuated motion of the proximal, middle, and distal phalanges; to improve adaptability through progressive self-wrapping when fruit-position deviation or partial enclosure occurs; and to reduce damage by removing talon-like contact and optimizing the contact geometry of the grasping area. Third, in the structural design section, we explain that the progressive multi-point closing mode distributes contact load more evenly and improves tolerance to fruit-size variation and positioning error, thereby reducing fruit slippage and local squeezing damage. Finally, in the Conclusion, we connect these design features with the experimental findings, particularly the lower separation force and the reduced plant/fruit damage observed in the integrated harvesting tests.

**Q2.** No direct correlation is demonstrated between these responses and harvesting success rate or fruit damage metrics. Also the optimization objective lacks multi-criteria justification.

Response: Thank you for this important comment. We have revised the manuscript as follows. First, in the Abstract, Introduction, and the "Response surface design" section, we now state explicitly that DCMP was selected to characterize the compressive clearance between the gripper and the fruit, which is directly related to squeezing damage risk, whereas DCDP was selected to characterize the distal enclosure space, which is related to retention stability during detachment and the probability of interference from pedicels, stems, or leaves. Second, we clarified that the target values of DCMP = 27 mm and DCDP = 41 mm were not arbitrary; they were set as a compromise based on the measured fruit size and repeated grasping observations, so that low compression damage and stable fruit retention could be satisfied simultaneously. Third, in the "Optimization results" and "Performance test" sections, we now explain more clearly that the field validation of the optimized end-effector (88% success rate for the pulling pattern and 91% for the integrated picking pattern) provides practical support for using DCMP and DCDP as optimization responses.

**Q3.** The manuscript mentions conducting 29 experiments but does not describe replication strategy or randomization details adequately.

Response: Thank you for this valuable comment. We have revised the manuscript to clarify the replication and randomization strategy of the 29 experiments. Specifically, we now state that the 29 experiments refer to the 29 runs of the 4-factor, 3-level Box-Behnken design used for structural-parameter optimization, rather than the later harvesting performance validation tests. We further clarify that the design was generated in randomized order in Design-Expert, and that it included five replicated center points (Runs 4, 6, 16, 27, and 29 in Table 2) to estimate pure error and evaluate lack of fit. In addition, for each BBD run, both response variables (DCMP and DCDP) were measured 10 times under the same parameter setting, and the average values were used for the subsequent regression and ANOVA. We also note in the harvesting validation section that the positions of cherry tomato plants within the cultivation layer were randomly altered and fruits at different heights were selected to reduce environmental and positional bias. These revisions have been highlighted in yellow in the revised manuscript.

**Q4.** Environmental factors such as fruit orientation, plant density, or stem stiffness were not incorporated into the design although they strongly influence robotic harvesting success.

Response: We sincerely thank the reviewer for this valuable comment. We agree that environmental factors such as fruit orientation, local plant density, and pedicel stiffness can significantly affect target localization, enveloping performance, separation resistance, and ultimately the success rate of robotic harvesting. In the present study, the primary objective of the RSM-BBD optimization stage was to identify how the key geometric parameters of the end-effector (WDMP, TDMP, AEDP, and DPAS) influence the gripping space and stable enveloping capability. Therefore, the optimization problem was intentionally defined as a structure-dominated model under relatively controlled conditions, so that the contributions of structural parameters to DCMP and DCDP could be isolated. For this reason, these environmental factors were not parameterized as independent variables in the Box-Behnken design. To make this research boundary clearer, we have added explicit statements in the revised manuscript. First, in the "Response surface design" section, we clarify that fruit orientation, local canopy density, and pedicel/stem stiffness were not parameterized as BBD factors in this study, but were treated as external harvesting conditions for the subsequent validation stage. Second, in the "Performance test" section, we explain that the validation experiments were conducted under real canopy conditions in a plant factory, where the optimized end-effector was exposed to different fruit positions, local occlusion partial enveloping situations, and variations in local canopy density. Third, in the "Conclusion" section, we explicitly state that these environmental factors should be incorporated into future work together with structural parameters for coupled optimization. Through these revisions, the manuscript now more clearly distinguishes the scope of structural parameter optimization from the external influencing factors present in real harvesting environments. This improves the clarity of the study boundaries, the applicability of the experiments, and the direction for future research.

**Q5.** The claim that tomato physical characteristics have minimal effect on picking success lacks sufficient experimental support.

Response: We thank the reviewer for this important comment and agree that the original wording was overly strong. In the revised manuscript, we removed the unsupported statement that tomato physical characteristics have minimal effect on picking success. Instead, we now clarify that the reported fruit diameter and mass were used only to define the tested sample range, and that fruits were deliberately selected at a similar maturity stage and within a relatively narrow size/mass distribution to reduce biological variability during the structural optimization stage. We further state that the present RSM-BBD optimization was designed to isolate the effect of end-effector geometry under controlled fruit conditions, whereas the independent influence of fruit physical characteristics on picking success or separation force was not quantified in this study. Accordingly, the validation results are now described as applying to the tested fruit-size range rather than implying universal insensitivity to fruit physical characteristics. We also added this point to the study limitations and future-work discussion.

**Q6.** Predicted R² values differ notably from adjusted R² values in one model, indicating possible overfitting or model instability. Residual diagnostics are presented only visually without statistical validation. External validation or confirmatory experiments using new datasets are missing.

Response: Thank you for this careful and constructive comment. We agree that the original presentation of model adequacy and validation was not sufficiently explicit. In the revised manuscript, we have addressed this issue from three aspects. First, in the “Variance Analysis” section, we corrected and clarified the interpretation of Pred. *R*² versus Adj. *R*². We now explicitly state that the DCMP model shows a lower Pred. *R*² (0.6956) than Adj. *R*² (0.8943), which indicates weaker out-of-sample predictive ability than the DCDP model rather than claiming that the two values are closely aligned. At the same time, we retained the corresponding R² and Adequate Precision results to show that the model still provides sufficient signal for parameter optimization within the studied factor space. Second, to supplement the visual residual plots, we added quantitative residual diagnostics. Specifically, we report Shapiro-Wilk test results for both fitted models. The DCMP residuals did not show a significant departure from normality, whereas the DCDP residuals showed mild non-normality. We therefore revised the manuscript to interpret the DCDP model with additional caution and to judge its adequacy jointly by ANOVA significance, residual trend, and confirmatory experiments rather than by visual inspection alone. Third, we clarified that the five experiments listed in Table 7 were used as confirmatory tests at parameter combinations outside the 29-run BBD fitting dataset. These additional experiments yielded prediction errors below 5%, with mean absolute percentage errors of 2.73% for DCMP and 1.97% for DCDP. We also retained the rounded practical verification experiment (16 mm, 5 mm, 90°, and 7.5 mm), which produced measured responses of 27 ± 0.67 mm and 41 ± 0.55 mm. These additions provide external confirmation that the fitted equations are reliable for optimization within the investigated design space.

**Q7.** The sample size and experimental replication for validation experiments are unclear. Fruit damage rate, harvesting speed, and operational reliability are not comprehensively evaluated. The results do not include comparisons under different field conditions.

Response: Thank you for this valuable comment. We have revised the manuscript to clarify the validation sample size and replication strategy, to make the practical performance metrics more explicit, and to better explain the scope and limitation of the field-condition comparison.

First, the experimental design and replication were clarified in the Materials and Methods section. We now explicitly state that the 29 runs correspond to the RSM-BBD structural-parameter measurement experiments, not the later harvesting performance validation tests. For each BBD run, both response variables (DCMP and DCDP) were measured 10 times and averaged for regression and ANOVA, and the 29-run design included five replicated center points (Runs 4, 6, 16, 27, and 29) to estimate pure error and evaluate lack of fit. In addition, five optimal solutions outside the 29-run fitting dataset were used as confirmatory tests to externally verify the fitted equations, followed by a practical verification experiment with the adjusted optimal parameter combination.

Second, the practical validation indicators were expanded and clarified. We now explicitly state that the optimized parameter combination was validated using harvesting success rate, plant damage, post-harvest fruit quality variation, single-fruit picking process time, single-fruit picking time, and maximum separation force (MSF). In the "Performance test" section, we further define the indicators and report the results for two harvesting modes. Specifically, the pulling pattern achieved an 88% harvesting success rate, a 17% plant damage rate, 20% and 35% post-harvest quality change rates at 72 h and 168 h, respectively, and an average single-fruit picking time of 4.81 s. The integrated picking pattern achieved a 91% harvesting success rate, a 3% plant damage rate, 11% and 22% post-harvest quality change rates at 72 h and 168 h, respectively, and an average single-fruit picking time of 6.08 s.

Third, we clarified the scope of the field-condition comparison. Because the RSM-BBD stage was intended to isolate structural effects, fruit size/mass and environmental variables such as fruit orientation, local canopy density, and pedicel stiffness were not parameterized in the response-surface model. However, in the plant-factory validation, the optimized end-effector was exposed to naturally varying fruit positions, partial occlusion states, and local canopy density. We have now stated this explicitly and also acknowledged in the Conclusion that the present study remains a geometry-centered optimization.

**Q8.** The manuscript occasionally mixes unit formatting. Mathematical symbols should be consistently defined when first introduced.

Response: Thank you for this observation. We carefully checked the manuscript and revised the presentation of units and symbols throughout the text and tables. We explicitly defined the mathematical symbols at their first appearance. In the "Response surface design" section, *X*_1_, *X*_2_, *X*_3_, and *X*_4_ are now clearly defined when first introduced.

**Q9.** The manuscript uses Design-Expert software but does not specify version justification or model selection rationale.

Response: Thank you for this helpful comment. We agree that the original manuscript did not sufficiently explain either the software choice or the rationale for selecting the response-surface model.

In the revised manuscript, we now explicitly state that the response-surface analysis was performed using Design-Expert 6.0.8. This version was selected because it provides a mature implementation of Box–Behnken design generation, quadratic response-surface fitting, ANOVA, and desirability-based multi-response optimization, which together match the statistical workflow adopted in the present study.

We also added the rationale for model selection. Because the study involved four structural factors at three levels and the objective was to evaluate possible curvature and two-factor interactions while keeping the experiment size manageable, a quadratic response-surface model based on a Box–Behnken Design (BBD) was selected as an efficient and appropriate framework for structural optimization. Compared with a full factorial design, BBD substantially reduces the number of required experimental runs while remaining suitable for estimating second-order effects. In addition, the response-surface stage was intended to extend the preceding single-factor screening by examining interaction and curvature effects among the selected key factors.

It is worth noting that the above modified parts are marked red in the revised manuscript.

Finally, special thanks to you for your good comments.

Reviewer #2:

We sincerely thank Reviewer #2 for the positive and encouraging comments on our manuscript. We are grateful that the reviewer recognized the novelty of the proposed bionic end-effector, the clarity of the initial theoretical and biomimetic design framework, and the effectiveness of the RSM-based statistical optimization.

As presented in the manuscript, the study developed a vulture-inspired bio-inspired phalangeal chain gripper to improve grasp stability, adaptability to partial enclosure, and reduction of fruit slippage and damage during cherry tomato harvesting. In addition, the RSM-BBD analysis successfully identified the optimal structural parameter combination (WDMP = 16 mm, TDMP = 5 mm, AEDP = 90°, and DPAS = 7.5 mm), and the corresponding validation experiments confirmed the effectiveness of the optimized design, with a harvesting success rate of at least 88% within the tested fruit-size range. These results support the reviewer’s assessment regarding both the conceptual clarity and the statistical validity of the work.

In addition to the specific responses to the academic editor and reviewers listed above, we have carefully proofread the entire manuscript to correct grammatical errors, improve sentence clarity, and ensure consistency in terminology. Furthermore, we have adjusted the formatting to comply with the journal’s requirements, including but not limited to reference style, heading hierarchy, figure and table captions, and line numbering. These changes do not affect the scientific content or the conclusions of the study.

Finally, we tried our best to improve the manuscript and made some changes in the manuscript. These changes will not influence the content and framework of the paper. We appreciate for reviewers’ warm work earnestly, and hope that the correction will meet with approval. Once again, thank you very much for the excellent and professional revision of our manuscript. Your careful review has helped to make our study clearer and more comprehensive. Those comments are all valuable and very helpful for revising and improving our paper. We have revised the manuscript accordingly, and our point-by-point responses are presented above.
