# Supplementary material for: Optimization of a bionic end-effector for automated cherry tomato harvesting using Response Surface Methodology and Box-Behnken design
Source: PLoS One. 2026 Aug 14;21(8):e0352701. doi: 10.1371/journal.pone.0352701 (PMC13475895; doi:10.1371/journal.pone.0352701)
Supplement: Peer review history

# Peer review: Decision Letter 0

**DOI:** 10.1371/journal.pone.0352701.r001

**Authors:** Debajyoti Kundu

10 Mar 2026

Dear Dr. Xie,

Thank you for submitting your manuscript to PLOS ONE. After careful consideration, we feel that it has merit but does not fully meet PLOS ONE’s publication criteria as it currently stands. Therefore, we invite you to submit a revised version of the manuscript that addresses the points raised during the review process.

==============================

**ACADEMIC EDITOR:**

Please submit your revised manuscript by Apr 24 2026 11:59PM. If you will need more time than this to complete your revisions, please reply to this message or contact the journal office at plosone@plos.org. When you're ready to submit your revision, log on to https://www.editorialmanager.com/pone/ and select the 'Submissions Needing Revision' folder to locate your manuscript file.

Please include the following items when submitting your revised manuscript:

- A letter that responds to each point raised by the academic editor and reviewer(s). You should upload this letter as a separate file labeled 'Response to Reviewers'.
- A marked-up copy of your manuscript that highlights changes made to the original version. You should upload this as a separate file labeled 'Revised Manuscript with Track Changes'.
- An unmarked version of your revised paper without tracked changes. You should upload this as a separate file labeled 'Manuscript'.

If you would like to make changes to your financial disclosure, please include your updated statement in your cover letter. Guidelines for resubmitting your figure files are available below the reviewer comments at the end of this letter.

If applicable, we recommend that you deposit your laboratory protocols in protocols.io to enhance the reproducibility of your results. Protocols.io assigns your protocol its own identifier (DOI) so that it can be cited independently in the future. For instructions see: https://journals.plos.org/plosone/s/submission-guidelines#loc-laboratory-protocols. Additionally, PLOS ONE offers an option for publishing peer-reviewed Lab Protocol articles, which describe protocols hosted on protocols.io. Read more information on sharing protocols at https://plos.org/protocols?utm_medium=editorial-email&utm_source=authorletters&utm_campaign=protocols.

We look forward to receiving your revised manuscript.

Kind regards,

Debajyoti Kundu, Ph.D

Academic Editor

PLOS One

Journal Requirements:

When submitting your revision, we need you to address these additional requirements.

1.Please ensure that your manuscript meets PLOS ONE's style requirements, including those for file naming. The PLOS ONE style templates can be found at

https://journals.plos.org/plosone/s/file?id=wjVg/PLOSOne_formatting_sample_main_body.pdf and

https://journals.plos.org/plosone/s/file?id=ba62/PLOSOne_formatting_sample_title_authors_affiliations.pdf.

2. Please note that PLOS One has specific guidelines on code sharing for submissions in which author-generated code underpins the findings in the manuscript. In these cases, we expect all author-generated code to be made available without restrictions upon publication of the work. Please review our guidelines at https://journals.plos.org/plosone/s/materials-and-software-sharing#loc-sharing-code and ensure that your code is shared in a way that follows best practice and facilitates reproducibility and reuse.

3. In your Methods section, please provide additional information regarding the permits you obtained for the work. Please ensure you have included the full name of the authority that approved the field site access and, if no permits were required, a brief statement explaining why.

4. Please be informed that funding information should not appear in the Acknowledgments section or other areas of your manuscript. We will only publish funding information present in the Funding Statement section of the online submission form. Please remove any funding-related text from the manuscript.

5. Please include captions for your Supporting Information files at the end of your manuscript, and update any in-text citations to match accordingly. Please see our Supporting Information guidelines for more information: http://journals.plos.org/plosone/s/supporting-information.

6. If the reviewer comments include a recommendation to cite specific previously published works, please review and evaluate these publications to determine whether they are relevant and should be cited. There is no requirement to cite these works unless the editor has indicated otherwise.

Additional Editor Comments (if provided):

The manuscript addresses an important topic in agricultural robotics and presents a bio-inspired end-effector optimized using RSM and Box–Behnken Design. However, several methodological clarifications, stronger statistical validation, and improved explanation of experimental design and harvesting performance metrics are required before the manuscript can be considered for publication.

[Note: HTML markup is below. Please do not edit.]

Reviewers' comments:

Reviewer's Responses to Questions

**Comments to the Author**

1. Is the manuscript technically sound, and do the data support the conclusions?

Reviewer #1: Yes

Reviewer #2: Yes

**********

2. Has the statistical analysis been performed appropriately and rigorously?

Reviewer #1: Yes

Reviewer #2: Yes

**********

3. Have the authors made all data underlying the findings in their manuscript fully available?

The PLOS Data policy

Reviewer #1: Yes

Reviewer #2: Yes

**********

4. Is the manuscript presented in an intelligible fashion and written in standard English?

Reviewer #1: Yes

Reviewer #2: Yes

**********

Reviewer #1: The manuscript presents the design and optimization of a bio-inspired end-effector for robotic cherry tomato harvesting using Response Surface Methodology (RSM) combined with Box-Behnken Design (BBD). The study is relevant and timely, considering increasing labour shortages and the growing need for agricultural automation. The integration of biomimetic design with statistical optimization is interesting and potentially valuable for precision harvesting robotics. The manuscript demonstrates experimental validation and presents quantitative results supporting the proposed design.

However, several methodological, statistical, and presentation-related concerns need to be addressed before the manuscript can be considered for publication. The major and minor comments are provided below.

1. The authors mention earlier bionic grippers and previous success rates but do not clearly explain how the present design fundamentally improves mechanical performance, adaptability, or damage reduction.

2. No direct correlation is demonstrated between these responses and harvesting success rate or fruit damage metrics. Also the optimization objective lacks multi-criteria justification.

3. The manuscript mentions conducting 29 experiments but does not describe replication strategy or randomization details adequately.

4. Environmental factors such as fruit orientation, plant density, or stem stiffness were not incorporated into the design although they strongly influence robotic harvesting success.

5. The claim that tomato physical characteristics have minimal effect on picking success lacks sufficient experimental support.

6. Predicted R² values differ notably from adjusted R² values in one model, indicating possible overfitting or model instability. Residual diagnostics are presented only visually without statistical validation. External validation or confirmatory experiments using new datasets are missing.

7. The sample size and experimental replication for validation experiments are unclear. Fruit damage rate, harvesting speed, and operational reliability are not comprehensively evaluated. The results do not include comparisons under different field conditions.

8. The manuscript occasionally mixes unit formatting. Mathematical symbols should be consistently defined when first introduced.

9. The manuscript uses Design-Expert software but does not specify version justification or model selection rationale.

This manuscript may consider for publication after incorporation of all suggestions.

Reviewer #2: The present article showed a novel approach to develop a bionic end-effector modulated automatic harvestor. The initial theory and biomimic approach is very clearly shown. In the later section, the RSM analysis proves the efficacy in statistical means and presents the optimized value.The article can be accepted in its form.

**********

what does this mean?). If published, this will include your full peer review and any attached files.

If you choose “no”, your identity will remain anonymous but your review may still be made public.

**Do you want your identity to be public for this peer review?**  For information about this choice, including consent withdrawal, please see our Privacy Policy

Reviewer #1: **Yes:**  Sukanta Dash, Senior Scientist, ICAR-IASRI, India

Reviewer #2: No

**********

[NOTE: If reviewer comments were submitted as an attachment file, they will be attached to this email and accessible via the submission site. Please log into your account, locate the manuscript record, and check for the action link "View Attachments". If this link does not appear, there are no attachment files.]

To ensure your figures meet our technical requirements, please review our figure guidelines: https://journals.plos.org/plosone/s/figures

You may also use PLOS’s free figure tool, NAAS, to help you prepare publication quality figures: https://journals.plos.org/plosone/s/figures#loc-tools-for-figure-preparation.

NAAS will assess whether your figures meet our technical requirements by comparing each figure against our figure specifications.

# Author comment: Author response to Decision Letter 1

**DOI:** 10.1371/journal.pone.0352701.r002

9 Apr 2026

Dear academic editor and reviewers,

Re: Manuscript ID: PONE-D-25-68743 and Title: Optimization of a bionic end-effector for automated cherry tomato harvesting using response surface methodology and box-behnken design.

Thank you for your letter and the valuable comments from both the editor and the reviewers concerning our manuscript entitled "Optimization of a bionic end-effector for automated cherry tomato harvesting using response surface methodology and Box-Behnken design" (PONE-D-25-68743).

Overall, the comments have been fair, encouraging and constructive, and we have learned much from them. We sincerely appreciate the constructive suggestions from the academic editor, as well as the insightful feedback from the reviewers. All of these suggestions have been carefully considered.

After carefully studying the reviewers’ comments and your advice, we have made corresponding changes to the paper. Based on the instructions provided in your letter, we have uploaded the file of the revised manuscript. Appended to this letter is our point-by-point response to the comments raised by the reviewers. The comments are reproduced and our responses are given directly afterward in a different color. We would like also to thank you for allowing us to resubmit a revised copy of the manuscript.

We would like to thank you for allowing us to resubmit a revised copy of the manuscript and we highly appreciate your time and consideration.

If you have any question about this paper, please don’t hesitate to let me know.

Sincerely.

Dr Xie.

Response to the academic editor's comments:

We very much appreciate the careful reading of our manuscript and valuable suggestions of the academic editor. We have carefully considered the comments and have revised the manuscript accordingly. The comments can be summarized as follows:

Q. The manuscript addresses an important topic in agricultural robotics and presents a bio-inspired end-effector optimized using RSM and Box–Behnken Design. However, several methodological clarifications, stronger statistical validation, and improved explanation of experimental design and harvesting performance metrics are required before the manuscript can be considered for publication.

Response: Thank you for this constructive summary. We have revised the manuscript to address each of the three major concerns—methodological clarity, statistical rigor, and experimental design explanation—as detailed below.

To address the request for methodological clarifications, we have explicitly stated in the Introduction, in the section "Structural design of bio‑inspired phalangeal chain gripper", and in the section "Response surface design" the improvement logic of the present vulture‑inspired end‑effector over our previous design: mechanical performance is enhanced through coordinated under‑actuated motion of the proximal, middle, and distal phalanges; adaptability is improved by progressive self‑wrapping under fruit‑position deviation; and damage is reduced by removing talon‑like features and optimising contact geometry. Furthermore, in the Abstract, in the Introduction, and in the "Response surface design" section, we have clearly explained why DCMP (representing compressive clearance and thus squeezing damage risk) and DCDP (representing distal enclosure space and thus fruit retention stability) were selected as response variables, and why their target values (27 mm and 41 mm) were chosen as a compromise based on measured fruit size and repeated grasping observations.

To meet the requirement for stronger statistical validation, we have supplemented the replication and randomisation details in the sections "Response surface design" and "Experimental method of cherry tomato harvesting": the 29 BBD runs were generated in randomised order by Design‑Expert, included five replicated centre points, and each response was measured ten times per parameter combination to estimate pure error and lack of fit. In the "Variance analysis" section, we have added normality test results (Shapiro‑Wilk p = 0.431 for DCMP and p = 0.013 for DCDP), and we now interpret the DCDP model with additional caution, supported by ANOVA significance and confirmatory experiments. Moreover, in the optimisation results subsection (following the variance analysis) and in the newly added Table 7, we have included five independent confirmatory runs (not part of the original 29 BBD runs), all with prediction errors below 5%, confirming the predictive reliability of the response‑surface equations.

To address the need for a clearer explanation of the experimental design and harvesting performance metrics, we have explicitly distinguished, in the sections "Experimental method of cherry tomato harvesting" and "Response surface design", the 29 BBD runs for structural‑parameter optimisation from the later harvesting performance validation tests to avoid confusion. In the "Performance test" section, we note that the field‑validated success rates of the optimised end‑effector (88% for the pulling pattern and 91% for the integrated picking pattern) provide practical support for using DCMP and DCDP as optimisation responses, while acknowledging that a direct regression between these responses and success rate/damage metrics has not been established here and should be addressed in future work. Finally, in the "Performance test" section and in the "Experimental method of cherry tomato harvesting" section, we have provided clearer definitions of plant damage, single‑fruit picking time, and maximum separation force (MSF), and explained how the integrated picking pattern reduces MSF and plant damage compared to the pulling pattern. We believe these revisions have substantially improved the clarity, rigour, and reproducibility of the manuscript.

Response to the reviewer's comments:

We are very grateful to your comments for the manuscript. According to your advice, we amended the relevant part in manuscript. All of your questions were answered one by one.

Reviewer #1:

We very much appreciate the careful reading of our manuscript and valuable suggestions of the reviewer. We have carefully considered the comments and have revised the manuscript accordingly. The comments can be summarized as follows:

Q1. The authors mention earlier bionic grippers and previous success rates but do not clearly explain how the present design fundamentally improves mechanical performance, adaptability, or damage reduction.

Response: Thank you for this valuable suggestion. In response, we revised the manuscript in several locations to make the improvement logic explicit. First, in the Introduction, we now state that the limitations of our earlier cherry tomato gripper were mainly related to stable enclosure and damage control during fruit detachment. Second, we clarify that the present vulture-inspired design was conceived to improve mechanical performance through coordinated under-actuated motion of the proximal, middle, and distal phalanges; to improve adaptability through progressive self-wrapping when fruit-position deviation or partial enclosure occurs; and to reduce damage by removing talon-like contact and optimizing the contact geometry of the grasping area. Third, in the structural design section, we explain that the progressive multi-point closing mode distributes contact load more evenly and improves tolerance to fruit-size variation and positioning error, thereby reducing fruit slippage and local squeezing damage. Finally, in the Conclusion, we connect these design features with the experimental findings, particularly the lower separation force and the reduced plant/fruit damage observed in the integrated harvesting tests.

Q2. No direct correlation is demonstrated between these responses and harvesting success rate or fruit damage metrics. Also the optimization objective lacks multi-criteria justification.

Response: Thank you for this important comment. We have revised the manuscript as follows. First, in the Abstract, Introduction, and the "Response surface design" section, we now state explicitly that DCMP was selected to characterize the compressive clearance between the gripper and the fruit, which is directly related to squeezing damage risk, whereas DCDP was selected to characterize the distal enclosure space, which is related to retention stability during detachment and the probability of interference from pedicels, stems, or leaves. Second, we clarified that the target values of DCMP = 27 mm and DCDP = 41 mm were not arbitrary; they were set as a compromise based on the measured fruit size and repeated grasping observations, so that low compression damage and stable fruit retention could be satisfied simultaneously. Third, in the "Optimization results" and "Performance test" sections, we now explain more clearly that the field validation of the optimized end-effector (88% success rate for the pulling pattern and 91% for the integrated picking pattern) provides practical support for using DCMP and DCDP as optimization responses.

Q3. The manuscript mentions conducting 29 experiments but does not describe replication strategy or randomization details adequately.

Response: Thank you for this valuable comment. We have revised the manuscript to clarify the replication and randomization strategy of the 29 experiments. Specifically, we now state that the 29 experiments refer to the 29 runs of the 4-factor, 3-level Box-Behnken design used for structural-parameter optimization, rather than the later harvesting performance validation tests. We further clarify that the design was generated in randomized order in Design-Expert, and that it included five replicated center points (Runs 4, 6, 16, 27, and 29 in Table 2) to estimate pure error and evaluate lack of fit. In addition, for each BBD run, both response variables (DCMP and DCDP) were measured 10 times under the same parameter setting, and the average values were used for the subsequent regression and ANOVA. We also note in the harvesting validation section that the positions of cherry tomato plants within the cultivation layer were randomly altered and fruits at different heights were selected to reduce environmental and positional bias. These revisions have been highlighted in yellow in the revised manuscript.

Q4. Environmental factors such as fruit orientation, plant density, or stem stiffness were not incorporated into the design although they strongly influence robotic harvesting success.

Response: We sincerely thank the reviewer for this valuable comment. We agree that environmental factors such as fruit orientation, local plant density, and pedicel stiffness can significantly affect target localization, enveloping performance, separation resistance, and ultimately the success rate of robotic harvesting. In the present study, the primary objective of the RSM-BBD optimization stage was to identify how the key geometric parameters of the end-effector (WDMP, TDMP, AEDP, and DPAS) influence the gripping space and stable enveloping capability. Therefore, the optimization problem was intentionally defined as a structure-dominated model under relatively controlled conditions, so that the contributions of structural parameters to DCMP and DCDP could be isolated. For this reason, these environmental factors were not parameterized as independent variables in the Box-Behnken design. To make this research boundary clearer, we have added explicit statements in the revised manuscript. First, in the "Response surface design" section, we clarify that fruit orientation, local canopy density, and pedicel/stem stiffness were not parameterized as BBD factors in this study, but were treated as external harvesting conditions for the subsequent validation stage. Second, in the "Performance test" section, we explain that the validation experiments were conducted under real canopy conditions in a plant factory, where the optimized end-effector was exposed to different fruit positions, local occlusion partial enveloping situations, and variations in local canopy density. Third, in the "Conclusion" section, we explicitly state that these environmental factors should be incorporated into future work together with structural parameters for coupled optimization. Through these revisions, the manuscript now more clearly distinguishes the scope of structural parameter optimization from the external influencing factors present in real harvesting environments. This improves the clarity of the study boundaries, the applicability of the experiments, and the direction for future research.

Q5. The claim that tomato physical characteristics have minimal effect on picking success lacks sufficient experimental support.

Response: We thank the reviewer for this important comment and agree that the original wording was overly strong. In the revised manuscript, we removed the unsupported statement that tomato physical characteristics have minimal effect on picking success. Instead, we now clarify that the reported fruit diameter and mass were used only to define the tested sample range, and that fruits were deliberately selected at a similar maturity stage and within a relatively narrow size/mass distribution to reduce biological variability during the structural optimization stage. We further state that the present RSM-BBD optimization was designed to isolate the effect of end-effector geometry under controlled fruit conditions, whereas the independent influence of fruit physical characteristics on picking success or separation force was not quantified in this study. Accordingly, the validation results are now described as applying to the tested fruit-size range rather than implying universal insensitivity to fruit physical characteristics. We also added this point to the study limitations and future-work discussion.

Q6. Predicted R² values differ notably from adjusted R² values in one model, indicating possible overfitting or model instability. Residual diagnostics are presented only visually without statistical validation. External validation or confirmatory experiments using new datasets are missing.

Response: Thank you for this careful and constructive comment. We agree that the original presentation of model adequacy and validation was not sufficiently explicit. In the revised manuscript, we have addressed this issue from three aspects. First, in the “Variance Analysis” section, we corrected and clarified the interpretation of Pred. R² versus Adj. R². We now explicitly state that the DCMP model shows a lower Pred. R² (0.6956) than Adj. R² (0.8943), which indicates weaker out-of-sample predictive ability than the DCDP model rather than claiming that the two values are closely aligned. At the same time, we retained the corresponding R² and Adequate Precision results to show that the model still provides sufficient signal for parameter optimization within the studied factor space. Second, to supplement the visual residual plots, we added quantitative residual diagnostics. Specifically, we report Shapiro-Wilk test results for both fitted models. The DCMP residuals did not show a significant departure from normality, whereas the DCDP residuals showed mild non-normality. We therefore revised the manuscript to interpret the DCDP model with additional caution and to judge its adequacy jointly by ANOVA significance, residual trend, and confirmatory experiments rather than by visual inspection alone. Third, we clarified that the five experiments listed in Table 7 were used as confirmatory tests at parameter combinations outside the 29-run BBD fitting dataset. These additional experiments yielded prediction errors below 5%, with mean absolute percentage errors of 2.73% for DCMP and 1.97% for DCDP. We also retained the rounded practical verification experiment (16 mm, 5 mm, 90°, and 7.5 mm), which produced measured responses of 27 ± 0.67 mm and 41 ± 0.55 mm. These additions provide external confirmation that the fitted equations are reliable for optimization within the investigated design space.

Q7. The sample size and experimental replication for validation experiments are unclear. Fruit damage rate, harvesting speed, and operational reliability are not comprehensively evaluated. The results do not include comparisons under different field conditions.

Response: Thank you for this valuable comment. We have rev

Attachment Submitted filename: Response to Reviewers.docx

# Editor report: Decision Letter 1

**DOI:** 10.1371/journal.pone.0352701.r003

**Authors:** Jianguo Wang

14 Jun 2026

Optimization of a Bionic End-Effector for Automated Cherry Tomato Harvesting Using Response Surface Methodology and Box-Behnken Design

PONE-D-25-68743R1

Dear Dr. Xie,

We’re pleased to inform you that your manuscript has been judged scientifically suitable for publication and will be formally accepted for publication once it meets all outstanding technical requirements.

Within one week, you’ll receive an e-mail detailing the required amendments. When these have been addressed, you’ll receive a formal acceptance letter and your manuscript will be scheduled for publication.

An invoice will be generated when your article is formally accepted. Please note, if your institution has a publishing partnership with PLOS and your article meets the relevant criteria, all or part of your publication costs will be covered. Please make sure your user information is up-to-date by logging into Editorial Manager at Editorial Manager® and clicking the ‘Update My Information' link at the top of the page. For questions related to billing, please contact billing support.

If your institution or institutions have a press office, please notify them about your upcoming paper to help maximize its impact. If they’ll be preparing press materials, please inform our press team as soon as possible -- no later than 48 hours after receiving the formal acceptance. Your manuscript will remain under strict press embargo until 2 pm Eastern Time on the date of publication. For more information, please contact onepress@plos.org.

Kind regards,

Jianguo Wang, PhD

Academic Editor

PLOS One

Additional Editor Comments (optional):

Reviewers' comments:

# Editor report: Acceptance letter

**DOI:** 10.1371/journal.pone.0352701.r004

**Authors:** Jianguo Wang

PONE-D-25-68743R1

PLOS One

Dear Dr. Xie,

I'm pleased to inform you that your manuscript has been deemed suitable for publication in PLOS One. Congratulations! Your manuscript is now being handed over to our production team.

At this stage, our production department will prepare your paper for publication. This includes ensuring the following:

* All references, tables, and figures are properly cited

* All relevant supporting information is included in the manuscript submission,

* There are no issues that prevent the paper from being properly typeset

You will receive further instructions from the production team, including instructions on how to review your proof when it is ready. Please keep in mind that we are working through a large volume of accepted articles, so please give us a few days to review your paper and let you know the next and final steps.

Lastly, if your institution or institutions have a press office, please let them know about your upcoming paper now to help maximize its impact. If they'll be preparing press materials, please inform our press team within the next 48 hours. Your manuscript will remain under strict press embargo until 2 pm Eastern Time on the date of publication. For more information, please contact onepress@plos.org.

You will receive an invoice from PLOS for your publication fee after your manuscript has reached the completed accept phase. If you receive an email requesting payment before acceptance or for any other service, this may be a phishing scheme. Learn how to identify phishing emails and protect your accounts at https://explore.plos.org/phishing.

If we can help with anything else, please email us at customercare@plos.org.

Thank you for submitting your work to PLOS ONE and supporting open access.

Kind regards,

PLOS ONE Editorial Office Staff

on behalf of

Dr. Jianguo Wang

Academic Editor

PLOS One
